# A Human Lung-Associated *Streptomyces* sp. TR1341 Produces Various Secondary Metabolites Responsible for Virulence, Cytotoxicity and Modulation of Immune Response

**DOI:** 10.3389/fmicb.2019.03028

**Published:** 2020-01-17

**Authors:** Andrej Herbrík, Erika Corretto, Alica Chroňáková, Helena Langhansová, Petra Petrásková, Jiří Hrdý, Matouš Čihák, Václav Krištůfek, Jan Bobek, Miroslav Petříček, Kateřina Petříčková

**Affiliations:** ^1^Institute of Immunology and Microbiology, 1st Faculty of Medicine, Charles University, Prague, Czechia; ^2^Institute of Soil Biology, Biology Centre Academy of Sciences of the Czech Republic, České Budějovice, Czechia; ^3^Faculty of Science, University of South Bohemia, České Budějovice, Czechia; ^4^Department of Chemistry, Faculty of Science, Jan Evangelista Purkyně University in Ústí nad Labem, Ústí nad Labem, Czechia

**Keywords:** *Streptomyces*, human pneumonia, pathogenicity, secondary metabolites, hemolysis, actinomycin, cytolytic polyenes

## Abstract

Streptomycetes, typical soil dwellers, can be detected as common colonizers of human bodies, especially the skin, the respiratory tract, the guts and the genital tract using molecular techniques. However, their clinical manifestations and isolations are rare. Recently they were discussed as possible “coaches” of the human immune system in connection with certain immune disorders and cancer. This work aimed for the characterization and evaluation of genetic adaptations of a human-associated strain *Streptomyces* sp. TR1341. The strain was isolated from sputum of a senior male patient with a history of lung and kidney TB, recurrent respiratory infections and COPD. It manifested remarkably broad biological activities (antibacterial, antifungal, beta-hemolytic, etc.). We found that, by producing specific secondary metabolites, it is able to modulate host immune responses and the niche itself, which increase its chances for long-term survival in the human tissue. The work shows possible adaptations or predispositions of formerly soil microorganism to survive in human tissue successfully. The strain produces two structural groups of cytotoxic compounds: 28-carbon cytolytic polyenes of the filipin type and actinomycin X2. Additionally, we summarize and present data about streptomycete-related human infections known so far.

## Introduction

Streptomycetes are commonly reported as soil bacteria with a life-style similar to fungi (Chater and Hopwood, 1993). Though indisputably based in soil, they can inhabit many more habitats including soil-like substrates (bat guano, tertiary sediments, and sea sediments), sea water and extreme habitats (arid, hypersaline or heavy metals-polluted biotopes, extreme temperatures, etc.) (Kampfer et al., 2014). Recently, numerous works report their important symbiotic relationships with plants and animals (Kaltenpoth et al., 2005; Behie et al., 2016). In some cases, they develop tight bonds with the host as it is documented in leaf-cutting (Haeder et al., 2009), but also other families of ants (Liu et al., 2018), and in the digging wasps. The wasp females form specific cultivation organs in their antennae to grow streptomycetes that in return produce antifungals protecting their offspring from deadly fungal diseases (Kaltenpoth et al., 2005; Nechitaylo et al., 2014). A recent work describes the first clearly documented case of their mutualism with vertebrates, sea turtles (Sarmiento-Ramirez et al., 2014). In almost all reported cases the streptomycetes protect the host or its food resources from pathogenic fungi.

In contrast, *Streptomyces* interaction with human seems to be marginal, or maybe neglected until recent days. Their ability to produce a plethora of secondary metabolites specifically targeted to human or mammalian cells is well known. Some of these have already been applied in human medicine as immunomodulators (rapamycin, tacrolimus) (Bolourian and Mojtahedi, 2018a) and cancerostatics (mitomycin C, bleomycin, actinomycin, doxorubicin and many others) (Olano et al., 2009). Despite this, only limited data report direct colonization of human bodies by streptomycetes. These include mainly endemic human streptomycetomas caused by *S. somaliensis* and *S. sudanensis* and rarely by other species in sub-Saharan Africa and India (Martin et al., 2004; van de Sande, 2013; Verma and Jha, 2019). It should be noted that even in the case of these “well-established” streptomycete pathogens, no specific virulence factors were recognized, and nothing is known about the molecular mechanisms of their pathogenicity. *S. somaliensis* has a remarkably small genome: 5.7 Mb compared to an average size of 7–10 Mbp of other streptomycetes. A reduced genome is a trend typical for obligatory, mostly intracellular pathogens (Kirby et al., 2012). In general, streptomycetes cause suppurative granulomatous tissue changes. The infection starts from the surface skin structures. If untreated, it proceeds to muscles, bones and may even spread via the lymphatic system or blood and cause a systemic disease. Compared to similar, fungi-originated, mycetomas, the actinomycetomas progress more rapidly and affect the deep bone structures in a short time. However, the antibiotic long-term treatment is quite successful (Relhan et al., 2017). Certain respiratory diseases (e.g., farmer’s lung disease) have been associated with inhalation of actinomycete spores, together with spores of fungi (Roussel et al., 2005; Cano-Jimenez et al., 2016). As streptomycete spores are significantly smaller than those of fungi (1.2–2.5 μm vs. 2.5–10 μm), they are extremely likely to reach the alveoli, which may elicit potential risk for exposed residents (Awad and Farag, 1999). Streptomycetes are often mentioned as etiologic agents of inflammatory diseases originated from water-damaged houses. The study of Huttunen et al. (2003) proves them to be one of the top microbial producers of pro-inflammatory and cytotoxic compounds in wet buildings. Scarce reports bring information of other infections of human manifested mainly as pulmonary infections, bacteremias and different organs abscesses (Kapadia et al., 2007) – see Supplementary Table S1 for details. Typical patients are immunocompromised, undergoing cancer therapy, etc., but also infections of immunocompetent people are reported (Yacoub et al., 2014).

For a long time, the presence of streptomycetes in healthy human microbiome remained neglected, though it was clearly reported in various animals. We suppose that their colonization of human tissues was and still is underestimated due to the lack of selective streptomycete cultivation techniques, their low growth rates and the generally accepted opinion of clinical microbiologist to view them as an air-born contamination. However, recent molecular data on the human microbiome confirm that they are present in the healthy skin (Gallo and Hooper, 2012), the gastro-intestinal tract (Bolourian and Mojtahedi, 2018b), the respiratory tract (Huang et al., 2015), and surprisingly also in the uterus (Collado et al., 2016). The major resource, whether ingested or inhaled, is soil, the contact with which is often mentioned as an important factor of human health (Sing and Sing, 2010). This makes them one of the hottest candidates as control agents of the developing microbial communities and coaches of the host immune system (Bolourian and Mojtahedi, 2018a, b). In fact, their huge variability of metabolite structures and activities must originate from broad interactions with various organisms, tissues and cells. They were recently also mentioned in connection to the suspected communication of gut microbiota with other organs, the gut-brain and gut-lung axes (Engevik and Versalovic, 2017; Bolourian and Mojtahedi, 2018b). Next, substantial changes in their abundance correlate with certain diseases or treatments (Huang et al., 2015; Wu et al., 2017; Silva et al., 2018). And last, we should also note that human guts contain considerably lower counts of streptomycetes than we can see in other, even closely related animals (Bolourian and Mojtahedi, 2018a). This was mentioned in connection to the typically cooked human diet, which destroys their important source – the soil particles-contaminated raw food. If we accept the theory of streptomycetes as one of the immune system coaches, their substantial reduction in human guts may correlate with the high incidence of the inflammatory bowel disease and gut cancer, almost unknown in animals (Rea et al., 2018). Taking all the recorded data together, the question arises: Can we consider streptomycetes to be friends or foes?

This work presents the characterization of *Streptomyces* sp. TR1341 strain isolated from the sputum of a patient with a history of multiple-organ TB, repeated respiratory infections and COPD. The patient was a senior man, living in the region with dust-polluted air. The strain was selected from our collection of about 80 strains of human specimen-originated streptomycetes due to a wide range of activities: from hemolysis to growth-inhibiting activities against both fungi and bacteria and other. We aimed to identify its specific genetic or metabolic features allowing it to colonize human tissues successfully. These human-adapted strains, in general, may serve as a great source of novel bioactive compounds specifically designed to modulate human cells behavior, e.g., suppressing the action of the immune cells to eliminate them. Moreover, they may substantially alter the behavior of the common microbiota and pathogens by targeted antibiotic activities or quorum quenching mechanisms (Park et al., 2005; Stubbendieck et al., 2016).

## Materials and Methods

### Cultivation Media and Microbial Strains

The streptomycete strain was cultivated in mannitol-soya (MS) agar without CaCl_2_ (Hobbs et al., 1989), Oatmeal agar (HiMedia) or standard Columbia blood agar with sheep blood (Oxoid) at 28°C. Pathogenic bacteria were cultivated in the Columbia blood agar in 37°C. Streptomycete liquid cultures for metabolite extractions were cultivated in standard GYM medium (Ochi, 1987), 200 rpm, 28°C.

Following microbial species were used in the activity assays: *Candida albicans* CCM 8186, *Staphylococcus aureus* DSM 346, *Bacillus subtilis* CCM 1718, *Streptococcus pneumoniae* CCM 4424, *Escherichia coli* DSM 682, *Klebsiella pneumoniae* DSM 681, and *Pseudomonas aeruginosa* DSM 50071 from the culture stock of the Institute of Immunology and Microbiology, 1st Faculty of Medicine, Charles University. *Staphylococcus aureus* MRSA, *Moraxella catarrhalis*, and *Neisseria pharyngis* were clinical isolates isolated in the Laboratory of Clinical Microbiology, General Teaching Hospital, Prague. *Saccharomyces cerevisiae* CCM 8191 and *Fusarium oxysporum* BCCO 20_0605 strains were provided from culture stock of Biology Centre Collection of Organisms (BCCO) at Institute of Soil Biology.

### Genome Sequencing and Assembly

DNA was extracted following the instructions of the Wizard Genomic DNA purification kit by Promega. An additional second centrifugation step was performed before resuspending the DNA. The sequencing of *Streptomyces* sp. TR1341 genome was performed by the Laboratory of Environmental Microbiology at the Institute of Microbiology, CAS, Prague. The library was prepared using the TruSeq PCR free LT library preparation kit (Illumina) and quantified with the KAPA library quantification kit (Roche). The library was sequenced using the Illumina MiSeq platform (Reagent kit v2, paired-end, 300 bp). Bowtie2 was used to screen for PhiX contamination (Langmead and Salzberg, 2012). Subsequently, reads quality was improved using Trimmomatic-0.36 (Bolger et al., 2014). Overlapping reads were merged using FLASH (Magoc and Salzberg, 2011). SPAdes 3.10.1 was used for assembly (Bankevich et al., 2012). The quality of the draft genome was assessed with QUAST (Gurevich et al., 2013) and Qualimap2 (Okonechnikov et al., 2016).

### Phylogenetic Analyses

Maximum likelihood phylogenetic tree of the 16S rRNA gene was calculated in RAxML-NG (Kozlov et al., 2019) with the GTR + F0 + G model: general time reversible model, optimized base frequencies by maximum-likelihood, gamma distribution and bootstrap 100. The 16S rRNA gene sequences of closely related strains were obtained from the NCBI database and are summarized in Supplementary Table S2. In addition, autoMLST (Alanjary et al., 2019) was used to perform the multi-locus taxonomy analysis using 85 single copy housekeeping genes (Supplementary Table S3).

### Hemolysis Assay

To assay the hemolysis, streptomycetes were inoculated from a sporulated culture on the MS agar onto the Columbia Blood agar. Development of hemolytic zones was observed in 2–5 days of cultivation at 28°C. The hemolysis type was evaluated according to the standard ASM guide (Blood Agar Plates and Hemolysis Protocols)^1^ : β-hemolysis (complete hemolysis) was defined as a complete lysis of red blood cells in the media around and under the colonies, the area appeared lightened (yellow) and transparent. Green or brown discoloration in the medium surrounding the colony, caused by the reduction of the red blood cell hemoglobin to methemoglobin, was assigned as α-hemolysis.

### Antibiotic Susceptibility Testing

The streptomycete strain was characterized for susceptibility to set of 21 antibiotics using a disk diffusion assay as follows (modification of Kirby-Bauer disk diffusion method). The antibiotic disks contained following antibiotics: amikacin 30, amoxicillin 25, amoxicillin + clavulanic acid 20 + 10, ampicillin 10, azithromycin 15, cefazolin 30, ceftriaxone 30, ciprofloxacin 5, clarithromycin 15, doxycycline 30, erythromycin 15, gentamicin 10, chloramphenicol 30, minocycline 30, ofloxacin 5, penicillin 6, rifampicin 5, streptomycin 10, tetracycline 30, trimethoprim-sulfamethoxazole 1.25 + 23.75, vancomycin 30. The numbers indicate amount of the antibiotics in μg per disk.

First, isolate was grown on Oat Meal agar (HiMedia Laboratories, India) for 14 days at 28°C, then spore suspension of an isolate was prepared in sterile tap water (McFarland density scale of 0.5) and 200 μL of spore suspension isolate was spread on the Mueller Hinton plates (Dulab, Czechia). Filter paper disks, containing selected antibiotics (Bio-Rad Laboratories, Hercules, CA, United States) were placed immediately after drying (no later than 15 min after plating) on the plates in triplicates. Inhibition zones were measured after 24 h of growth at 28°C. Simultaneously, *Staphylococcus aureus* DSM 346 was analyzed as internal quality control. Evaluation was done according to EUCAST guidelines for aerobic actinomycetes (e.g., *Corynebacterium*, *Nocardia*) or based on arbitrary breakpoints according to zone size distributions amongst strains in our collection (Adámkova V., personal communication).

### Cocultures With Pathogens

Due to the slower growth of streptomycetes, TR1341 was inoculated 48 h in advance and cultivated at 28°C. The pathogen line in a T-shape was added afterward. The vegetative growth of streptomycetes was already obvious, though they were usually not forming aerial mycelia yet. The bacteria were cocultured for next 1–2 days at 37°C and their interactions were observed.

### Cocultures With Human Macrophages

Human monocyte cell line THP-1 (purchased from the American Type Culture Collection, ATCC^®^ TIB-202^TM^) was cultured in the RPMI-1640 medium supplemented with 10% fetal calf serum (FCS), L-glutamine (292 μg/ml), penicillin G (100 U/ml), streptomycin (100 μg/ml; all from Biowest) and 50 μM mercaptoethanol (Sigma-Aldrich). Cells were cultured at 37°C in 5% CO_2_ and 95% air in a humidified incubator and passaged twice a week.

For the coculture experiments, the cells were resuspended in PBS with 1% FCS in concentration of 1 × 10^6^ cells per ml. Aliquots of 100 μl (10^5^ cells) were mixed with 10 μl of streptomycete spore suspension containing 10^6^ spores. In the negative control the same volume of PBS with 1% FCS was used instead of the spores, in the positive control 10 μl PMA (phorbol 12-myristate 13-acetate) was used to activate monocytes. The activation of THP-1 was performed using the FagoFlowEx^®^ kit (Sigma) according to manufacturer’s protocol. The cells were cocultured for 30 and 90 min at 37°C. Just before the flow cytometry analysis, the cells were washed once in PBS with 1% FCS and 1 μl of propidium iodide (PI, 50 μg/ml) was added to stain dead cells (monitoring of cytotoxicity). Respiratory burst of THP-1 cells was measured as the fluorescence of rhodamine 123 detected in 525 nm channel in BD FACS Canto II flow cytometer and analyzed using BD FACS Diva software. Data are expressed as average (*n* = 3) proportion of dead cells, dead but activated cells, neither activated nor dead cells and live activated cells. To compare the differences between control (PBS) and treated (TR1341) groups, two-way analysis of variance (ANOVA) followed by Bonferroni *post hoc* test in GraphPad Prism, version 5.0 was used. *P* ≤ 0.05 was considered as the level of statistical significance.

For the analysis of the coculture effect on the TR1341 secondary metabolome, the TR1341 spores (10^8^ cells/ml, 0.5 ml) were subjected to 10 min 55°C heat shock to induce their germination and transferred to 30 ml of RPMI-1640 media without antibiotics in a baffled Erlenmeyer flask. After 6 h of cultivation at 28°C, 200 rpm, 50 ml of RPMI-1640 with or without THP-1 cells (10^5^ cells/ml) and the cultivation continued for another 48 h. 8 ml of the culture was used as a seed to inoculate 80 ml of GYM. Fermentation in GYM continued in standard conditions for 3 days and secondary metabolites were extracted.

### Extraction of Secondary Metabolites

Secondary metabolites were extracted after 3 days of cultivation in 80 ml of GYM media in 500 ml baffled Erlenmeyer flask at 28°C, 200 rpm. The 8 ml inoculum for the fermentation culture was grown for 2 days under the same conditions. Two techniques were used to extract the secondary metabolites: SPE columns and organic solvent extraction. SPE columns were used solely for pilot extraction for analytical reasons. Subsequent organic solvent extraction provided sufficient extract amounts for the activity assays.

Pilot screening of the secondary metabolites production was done using SPE columns small-scale isolation using Oasis HLB 3cc 60 mg cartridge (hydrophilic-lipophilic balanced sorbent, Waters, United States) as described previously (Cihak et al., 2017).

Medium scale extraction was performed as liquid phase extraction using organic solvents. The 3 days fermentation culture (80 ml) was spun down: the medium and the cell pellet were processed separately. The cells were extracted with 1/2 volume of acetone for 30 min at 4°C, in a reciprocal shaker, and 250 rpm. The organic fraction was evaporated under 140 mbar pressure at 37°C. The rest of the liquid was extracted with 8 ml of ethylacetate using the same extraction conditions. NaCl was added in the post-fermentation media to 5M concentration and it was extracted with 1/3 volume of ethylacetate, the same conditions as above. Both ethylacetate extracts were combined, dried and dissolved in 200 μl of chloroform.

### LC Analysis of the Extracts

The pilot TLC fractionation of the extracts was performed using Silica gel TLC plates with 254 nm fluorescence indicator (Sigma-Aldrich, St. Louis, MO, United States). Ten μl of an extract were applied and developed in benzene: acetone (3: 2). The plates were recorded under short- and long-wavelength UV illumination (254 and 366 nm). LC-MS analysis was performed as described by Cihak et al. (2017).

### Bioactivities of the Metabolic Extracts

Hemolytic activity was assayed using standard Columbia blood agar. Ten μl of the culture metabolic extract dissolved in chloroform were dropped on the blood agar and let air-dry. The same amount of the pure chloroform was used as a negative control. The plate was incubated overnight at 37°C.

For the antibacterial and antifungal activity assays of the extracts, 10 μl was fractionated using TLC. The TLC plate was air-dried and printed on top of the blood agar previously covered with a cell suspension of the selected target microorganism as listed in Methods (McFarland density scale of 0.5). The TLC plate was left attached for 15 min to allow diffusion of the active compounds into the agar, removed and the plate was incubated overnight at 37°C for the development of the growth inhibition zones.

The cytotoxicity of the extracts was assayed using the THP-1 human monocyte cell line. For the human cell assays the streptomycete metabolic extracts were dissolved in DMSO. The concentration of the THP-1 cells used in the assay was 10^6^ cells/ml in RPMI-1640 with antibiotics. Final dilution of the extracts in the assay ranged from 1:500 to 1:50,000. The cells were cultured for 24 h at 37°C and 5.7% CO_2_. DMSO served as a negative control. Trypan Blue was used to stain and count the dead cells after the incubation.

The immunomodulatory effect of the extracts was assayed as their effect on THP-1 activation measured as production of the pro-inflammatory IL-1β by ELISA kit (Human IL-1beta Uncoated ELISA Kit, Invitrogen) according to the manufacturer’s recommendation. Non-lethal concentrations of the extract were used based on the cytotoxicity assays results. Results are presented as a mean of three independent experiments with standard error mean. *T*-test was employed to evaluate statistically significant differences between groups.

### Disruption of the Filipin Gene Cluster

Genomic DNA of streptomycetes was isolated using the Wizard Genomic DNA Extraction Kit (Promega) using standard manufacturer’s protocol for Gram-positive bacteria. For the disruption of the putative filipin gene cluster, the pGM160 plasmid with the temperature—sensitive replication was used (Muth et al., 1989). The disruption cassette contained the spectinomycin-resistance gene with conjugative *oriT* of the pIJ778 plasmid (Gust et al., 2004) surrounded by two arms homologous to the regions upstream and downstream of the five PKS I-encoding genes of the filipin cluster. The left arm (1050 bp) was amplified by PCR using FIL1L (5′-CAGCATGTTGGTGGTGGTCT-3′) and FIL1R (5′-CTCGACCATTTGCACTCCAC-3′) primers. The right arm (1299 bp) was amplified using FILCRO1L (5′-CTTTACGAAATCGGCGAGA-3′) and FILCRO1R (5′-GCTCGGACATGACTCTCCTT-3′) primers. The scheme of the insertion cassette is shown in Results. The cassette was cloned in the pGM160 vector and the resulting pFILDIS was introduced in *E. coli* ET12567/pUZ8002 by electroporation. From the resulting strain the construct was inserted in TR1341 by conjugation as described by Gust et al. (2004). The presence of the plasmid was checked by PCR using the FIL1L and spec200 (5′-ATTTTGCCAAAGGGTTCGTG-3′) primers and next using spec1300 (5′-TCACCAAGGTAGTCGGCAAA-3′) and FILCRO1R. Both spec primers anneal inside the spectinomycin resistance region of the cassette. The recombinant strain was cultured in YEME media supplemented with spectinomycin (400 μg/ml) in a rotary shaker at 28°C to late exponential phase and then the cultivation continued in the non-permissive temperature of 39°C. The culture was plated in serial dilutions on the MS plates with spectinomycin and MS plates with thiostrepton. Already after 2 days of non-permissive culture only spec^R^ thio^*S*^ colonies were found, indicating for the double crossing-over. The mutant colonies were checked using PCR with FIL1F – spec200 primers and spec1300 – FILCRO1R – both with positive band of a proper size and FIL1F – FILwtR (5′-GCGGTACGTCGGTGATCG-3′) and FILwtF (5′-TACTCGATGCTGGAGGACCA-3′) with no band formed in the disruption mutant. Both FILwt primers map within the deleted region of the filipin gene cluster. The parental *wt* strain was used as a control with no bands with spec primers and proper band size with FILwt primers.

## Results

### Strain Isolation and Identification

The strain was isolated in the District Hospital in Pribram, Czech Republic, 2008, from sputum of an 81-year old male patient with previous history of lung and kidney TB. The patient’s medical records also report ischemic heart disease, hypertension, gastroduodenal ulcer, lung fibrosis, COPD and repeated infections of the respiratory tract. He lived in a region with a high air pollution and died 4 years later due to the secondary liver cancer combined with pneumonia.

The sputum was subjected to selective cultivation for mycobacteria due to the suspected tuberculosis by a standard procedure (decontaminated sputum). This eliminated majority of microbiota with only mycobacteria resistant enough to the harsh treatment. To certain extent, streptomycetes can survive it as well, though with much lower rates. So, the streptomycetes can be isolated only from heavily colonized specimen. The strain was deposited in the collection of the National Reference Laboratory for Pathogenic Actinomycetes in the Local Hospital in Trutnov, Czech Republic, and kindly provided for our research.

**FIGURE 1:**
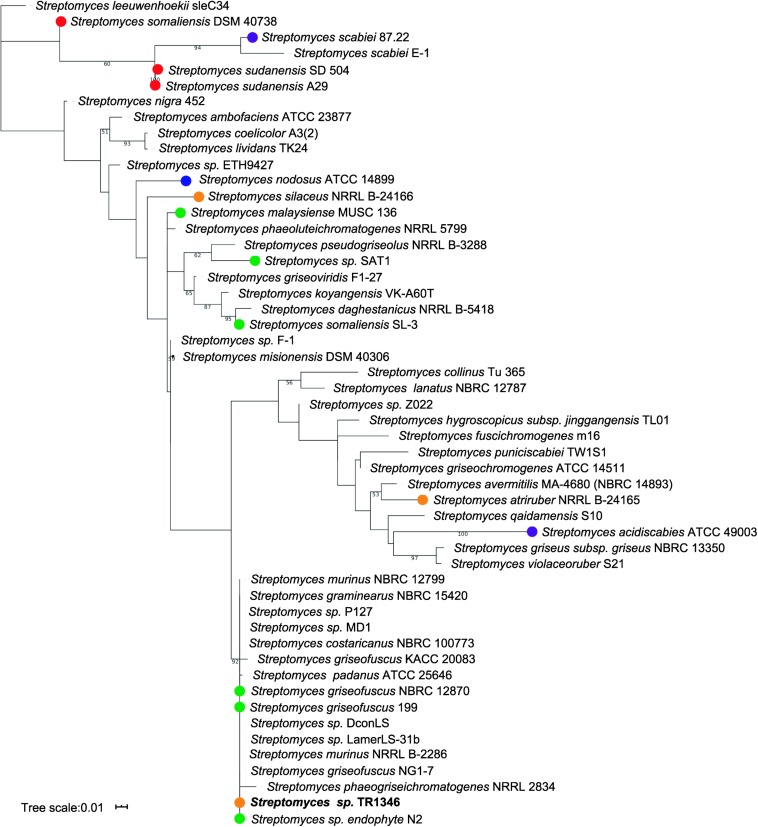
Maximum likelihood phylogenetic tree based on 16S rRNA gene. Only bootstrap values higher than 50 are shown. *Streptomyces* strains marked with dots were isolated from water (blue) or are plant associated (green), plant pathogens (purple), animal associated (orange) or human pathogens (red); all other strain were either isolated from soil or their isolation source could not be found in literature.

The only organism cultivated from the sputum had a typical filamentous appearance and grew in streptomycete-like colonies with gray-pigmented spores and yellow pigment produced to the culture media. The analysis of the 16S rRNA gene using EzTaxon (Chun et al., 2007) revealed *Streptomyces costaricanus* NBRC100773 as the closest match (1480/1480, 100%) – see Figure 1. AutoMLST (Alanjary et al., 2019) was used to perform the multi-locus taxonomy analysis using 86 single copy housekeeping genes (Supplementary Table S3 and Supplementary Figure S1). Both phylogenetic trees (Figure 1 and Supplementary Figure S1) suggest that *Streptomyces* sp. TR1341 belongs to the *Streptomyces murinus* group (clade 12 of family *Streptomycetaceae*), together with *S. griseofuscus* and *S. costaricanus* (Labeda et al., 2012)^2^. It is quite distant from plant and human pathogenic strains like *S. scabiei*, *S. sudanensis*, and *S. somaliensis* (Figure 1).

*Streptomyces* sp. TR1341 showed susceptibility to aminoglycosides, macrolides, vancomycin, 2nd generation of tetracyclines, chloramphenicol and aminopenicillin augmented with β-lactamase inhibitor. On the other hand, strain TR1341 was resistant to penicillin, ampicillin, cephalosporins, quinolones, rifampicin, tetracycline and trimethoprim with sulfamethoxazole (Supplementary Table S4).

The genomic data have been deposited under the following accession numbers: BioProject PRJNA558635, BioSample SAMN12496210, SRA SRR9903270 and WGS VSDL00000000.

### Hemolytic Activity

**FIGURE 2:**
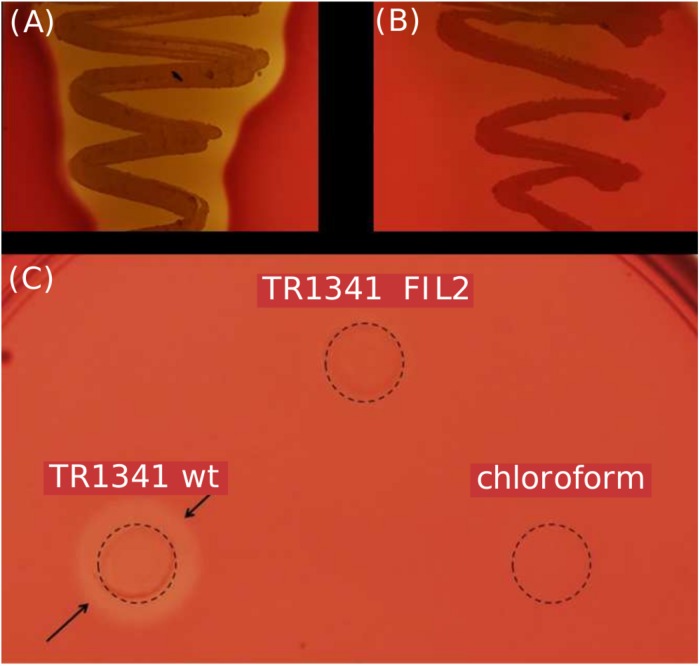
β-hemolytic features of *S. sp.* TR1341. **(A)** β-hemolysis of TR1341 wt colonies in a standard blood agar plate, 3 days incubation in 28°C. **(B)** Disruption of the filipin/fungichromin production in TR1341ΔFIL2 causes complete loss of β-hemolysis. Grown for 4 days at 28°C in blood agar. **(C)** Production of hemolytic secondary metabolites by the strain (TR1341 *wt*) – extracted from 3-days old culture grown in GYM media by acetone/ethylacetate liquid phase extraction. The amount of extract applied (10 μl) corresponds to 4 ml of original culture. Dashed circle indicates the extract drop border, the arrows show the hemolytic zone. TR1341ΔFIL2 is the extract of filipin-deficient mutant of TR1341, chloroform stands for the negative control (the solvent only). Photographed after 16 h incubation at 28°C.

The TR1341 strain possesses strong β-hemolytic activity (i.e., causing complete hemolysis) that can be observed using Columbia Blood Agar plates already after 48 h of cultivation at 28°C (Figure 2A). The activity is not so common in soil-originated strains of streptomycetes, though the exact rates have not been studied yet. A pilot screening of our collection of 102 human-associated strains revealed 77.5% of β-hemolytic strains, but only 56% among 184 soil-originated strains (data not shown). The hemolysis is considered to be one of the virulence factors of pathogens and typically results from the action of protein hemolysins of various types (Vandenesch et al., 2012).

As the soil-originated, α-hemolytic (i.e., viridating, incompletely hemolytic) *S. coelicolor* A3(2) type strain, the genome of TR1341 contains 4 putative homologs of known hemolysin genes (Table 1). Of these, SCO1782 is implicated in the α-hemolysis in *S. coelicolor* M145 (Rajesh et al., 2013). These genes seem to cluster with other putative genes that could play a role in the organism survival in the host tissue or even in intracellular parasitism. As an example we can mention ABC-transporter genes specific for Fe3^+^-siderophore just next to the SCO1782 homolog in TR1341 (see Table 1 for details and references).

**TABLE 1:** Putative hemolysin genes in TR1341 genome and their homologs in *S. coelicolor* A3(2) genome (NC_003888.3).

**TR1341 ORF**	***S. coelicolor* homolog**	**Similar to**	**Surrounding genes relevant to putative survival in the host**
FSY75_36865	SCO1782	**Bifunctional tRNA methyl-transferase/TlyA hemolysin** of *Mycobacterium tuberculosis* (AQO55200.1) and *Brachyspira* (former *Treponema) hyodysenteriae* (APP13931.1)	Fe3^+^-siderophore ABC-type transporter (Rahman et al., 2010; Forrellad et al., 2013; Rahman et al., 2015)
FSY75_17865	SCO2534	**HlyC/CorC family magnesium and cobalt efflux protein – TlyC family**: hemolysin C of *Brachyspira hyodysenteriae* (ACN83901.1) and Co^2+^-resistance protein CorC of *Salmonella typhimurium* (TKE78380.1), TlyC hemolysin of *Rickettsia prowazekii* (CAA72456.1)	Adenosine deaminase (Lee and Yilmaz, 2018) Methalo beta-lactamase (Somboro et al., 2018) Heat shock-inducible repressor and DnaJ chaperone (Forrellad et al., 2013)
FSY75_04245	SCO3882	**HylA/YidD**, *Aeromonas hydrophila* **β-hemolysin** (WP_011707926.1)	Thioredoxin, thioredoxin reductase (May et al., 2019)
FSY75_26430	SCO4978	**YqfA family hemolysin III family**, e.g., *Escherichia coli* (ANK03204.1)	Aspartate aminotransferase (Wang et al., 2016) Phosphoenolypyruvate carboxykinase (Forrellad et al., 2013) Thymidylate kinase (Challacombe et al., 2014)

*Gene Bank Accession numbers of homologous proteins given in brackets. The last column of the table shows genes that are encoded in the close vicinity of the putative hemolysin genes and may play roles in the bacteria survival in the host tissue. Protein family names are shown in bold.*

### Production of Siderophores

Siderophores are commonly produced by all bacteria: free-living, commensal and pathogenic. Iron is considered an essential element. However, it is not always freely available. For instance, concentrations of free ferric cations in human tissues are extremely low. Successful colonizers must develop efficient systems of Iron acquisition, where to seize the entire iron-chelating complexes of the host seems beneficial (Miethke and Marahiel, 2007). Genes putatively encoding such factors can be found in the close vicinity of the β-hemolytic gene homolog SCO1782 (Table 1).

### Filipin-Like Biosynthetic Gene Cluster Is Encoded in the Genome

The putative hemolysin genes described above can be identified in majority of available streptomycete genomes, too, disregarding their hemolytic activity (data not shown). No homologs of genes encoding typical β-hemolysis-causing hemolysins, such as streptolysins of the group A streptococci (Molloy et al., 2011), are present. Because if this, we speculated that the activity may not be caused by a protein factor, but a secondary metabolite. It has been well documented in the literature that certain polyene secondary metabolites of actinomycetes and fungi show hemolytic or general cytolytic properties: filipin- or pentamycin-type compounds with smaller rings and amphotericin- or candicidin-like compounds with larger rings. The smaller compounds cause quite harsh damage to the erythrocyte membranes, whereas the larger make subtler changes depending on many other factors. All these compounds are fungicidal and those with lower toxicity are used in clinical medicine – amphotericin B, nystatin A1, pentamycin – as crucial anti-fungal and anti-protozoal agents (Knopik-Skrocka and Bielawski, 2002).

**FIGURE 3:**
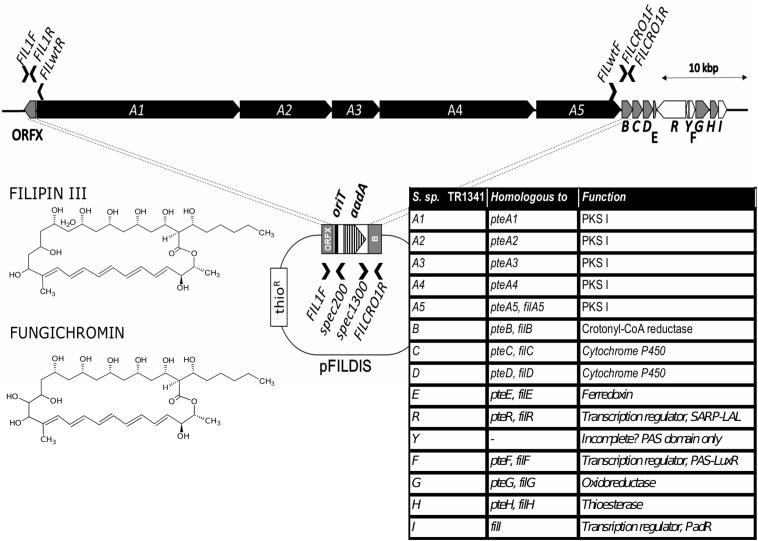
Filipin biosynthetic gene cluster of TR1341. The PKS I genes shown in black, tailoring genes in gray, regulatory in white. The gene annotation indicated in the table on the right and both compounds produced shown on the left. The PKSI genes disruption scheme shown in the middle A1-A5 genes replaced with the *aadA* spectinomycin resistance gene in TR1341ΔFIL2. Primers used to verify the gene disruption indicated by black arrows.

Scanning of the TR1341 genome revealed a putative gene cluster encoding filipin-type of pentaene compounds. All biosynthetic genes are similar to those of the known filipin producers *S. filipinensis* (Payero et al., 2015) and *S. avermitilis* (Vicente et al., 2014). The PKS I-encoding genes A1-A5 resemble those of filipin and encodes a 28-carbon macrolactone ring typical for filipin, fungichromin, antifungalmycin or thailandin. Genes B, C, D, E, G, and H encode chain-tailoring enzymes (Figure 3). The cluster encodes the same three regulatory genes as in *S. filipensis*: FilR, a SARP-LAL regulator; FilF, a regulator from the PAS-LuxR family, which is involved in the regulation of virulence factors in pathogenic actinobacteria (Ryan et al., 2015) and finally FilI, a PadR family transriptional regulator (Santos et al., 2012; Vicente et al., 2014). An extra, perhaps incomplete, ORF encodes a short protein with another PAS domain.

### The TR1341 Strain Produces Filipin III and Fungichromin

**FIGURE 4:**
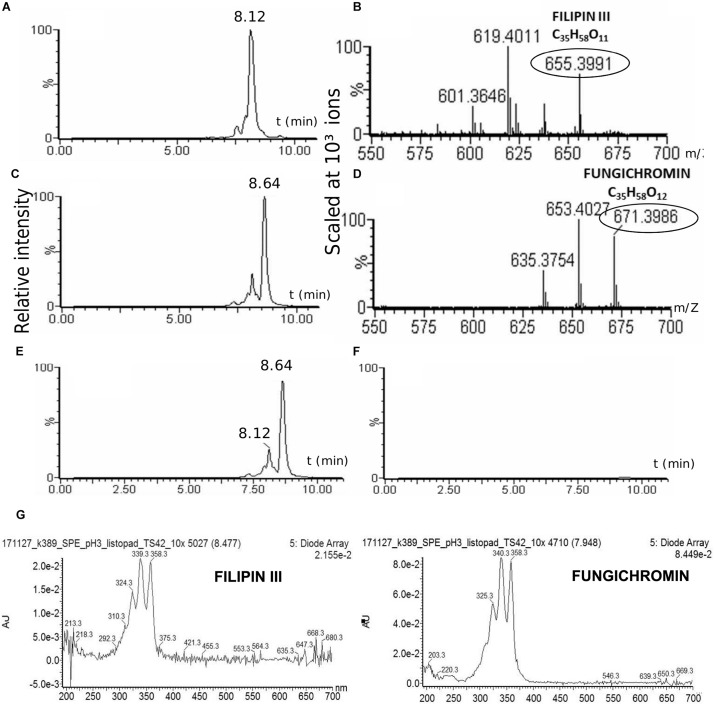
Production of cytolytic polyenes by TR1341. LC-MS analysis of the TR1341 extract of secondary metabolites: Filipin III **(A)** and fungichromin **(C)** peaks and corresponding MS analyses **(B,D)**. Disruption of the filipin III and fungichromin production in the TR1341ΔFIL2 mutant: **(E)** *wt* chromatogram, **(F)** mutant chromatogram. The absorption spectra of compounds correspond to the standards of both polyenes – 5 conjugated double bonds (Castanho et al., 1992) **(G).**

The strain was cultured in GYM liquid media and extraction of secondary metabolites was done following two methods: using solid phase extraction (SPE) 3 ml columns; liquid phase extraction using acetone and ethylacetate sequentially for the mycelia and ethylacetate only for the post-fermentation media. The extract was analyzed using UHPLC-MS. Both extracts contain two polyene compounds: filipin III and fungichromin in concordance with the genetic information (Figure 4). The extract itself showed β-hemolytic features: a quantity corresponding to 4 ml of initial culture formed a clear hemolytic spot on the blood agar plate after incubation at 37°C for 12 h (Figure 2C).

### 28-Carbon Polyenes Are the Only Factors Causing β-Hemolysis of TR1341

To verify the hemolytic activity of filipin and fungichromin in the TR1341 extract, a mutant strain unable to synthesize this type of compounds was designed. The entire region encoding all 5 PKS I genes was replaced with a cassette harboring the spectinomycin resistance gene (*aadA*) originated from the pIJ778 vector of the streptomycete Lambda Red system (Gust et al., 2004) using a temperature-sensitive pGM160 replicon (see in section Materials and Methods). Thio^R^ and spec^R^ recombinant colonies of putative filipin mutants were checked using PCR for a proper replacement (see Materials and Methods for details). The selected clone was designated as TR1341ΔFIL2. In order to exclude any contamination with the wt parent, the strain spores were collected and plated to grow in single colonies in MS media. A single spore (single colony) offspring was used in following experiments.

All the mutant colonies completely lost the β-hemolytic features (Figure 2B). However, incomplete hemolysis (viridation), seen as a greenish halo around the colonies was retained.

The TR1341ΔFIL2 mutant clones was subjected to regular fermentation. The extract was analyzed with UHPLC-MS and revealed no production of any of the cytolytic polyene macrolactones. The extract was assayed for the β-hemolytic activity together with the extract of the parental *wt* TR1341 strain. The hemolytic activity of the extract completely disappeared after disruption of filipin and fungichromin production (Figure 2C). This means that the hemolytic features of the strain are caused solely by the production of low-molecular weight hemolysins, the polyene secondary metabolites, and not by any other protein-type hemolysins putatively encoded in the genome.

### TR1341 Influences Growth and Virulence of Human Pathogens

**FIGURE 5:**
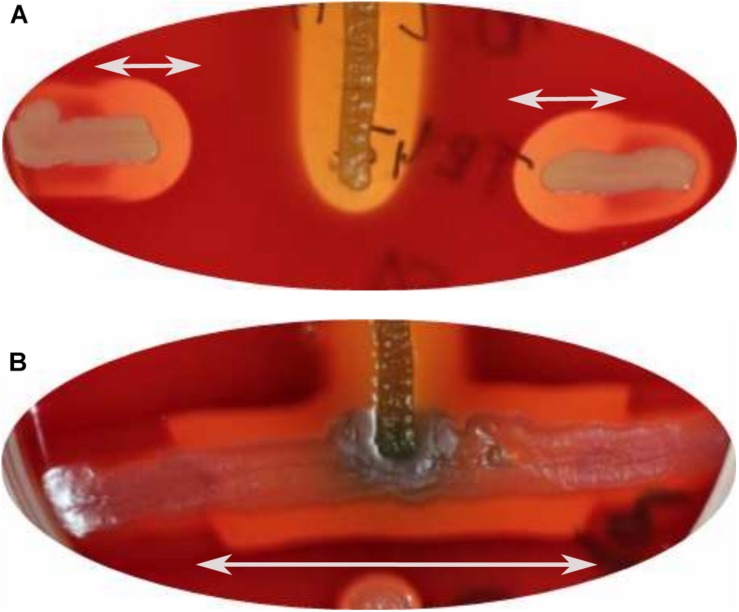
Interactions of TR1341 with human bacterial pathogens *in vivo*. Vertical line = TR1341, horizontal line inoculated in a T-shape to touch the TR1341 inoculation = the pathogen. **(A)** *Staphylococcus aureus:* Growth inhibitory zone *+* induction of β-hemolysis by TR1341 (white arrows). **(B)** *Pseudomonas aeruginosa:* Induction of β-hemolysis (white arrows), motility and colored secondary metabolites production in *Pseudomonas* in the close vicinity of TR1341.

In order to check the antibiotic activities of TR1341, first, the T-shape co-inoculation of TR1341 with selected human pathogens was performed and the appearance of a growth-inhibiting zone around TR1341 was recorded. The results are summarized in Table 2 and Figure 5. Next, the metabolic extracts of TR1341 and the TR1341ΔFIL2 mutant were fractionated by TLC and the chromatographic plate was directly “printed” on the top of a blood agar plate inoculated with the pathogens.

**TABLE 2:** Antibiotic activities of the TR1341 strain and the filipin/fungichromin mutant TR1341ΔFIL2.

**Pathogen**	**Growth inhibition**		**Other effects**
	**wt**	**TR1341ΔFIL2**	
*Candida albicans* CCM 8186	+	–	–
*Sacharomyces cerevisiae* CCM 8191	+	–	–
*Fusarium* sp. BCCO 20_0605	+	–	–
*Staphylococcus aureus* DSM 346	++	++	Hemolysis induced (only in the wt)
*Staph. aureus* MRSA (clinical)	+	+	n.d.
*Bacillus subtilis* CCM 1718	+	+	–
*Streptococcus pneumoniae* CCM 4424	+	++	Formation of a capsule inhibited
*Moraxella catarrhalis* (clinical)	+	+	–
*Neisseria pharyngis* (clinical)	+	++	–
*Escherichia coli* DSM 682	–	–	–
*Klebsiella pneumoniae* DSM 681	–	–	–
*Pseudomonas aeruginosa* DSM 50071	–	–	Hemolysis induced (only in the wt)

*Growth inhibition zone of the pathogen indicated: “+”: a small inhibitory zone < 8 mm; “++”: a big inhibitory zone > = 8 mm; “−“: no growth inhibition; n.d., not defined. Other effects on the growth and characteristics given in the last column. For a typical picture see the Supplementary Figure S2.*

The TLC fractionation-based assay of the wt and mutant has shown that TR1341 produces at least two bioactive compounds: the fungicidal one with hemolytic features and an antibacterial compound. For the fungicidal activities, only the macrolactone polyenes of filipin and fungichromin are responsible. The activity completely disappears in the TR1341ΔFIL2 mutant. Antibacterial activity has a different retention factor using the TLC system and has quite a wide spectrum of activities – it acts against all assayed Gram-positives, including methicillin-resistant *Staphylococcus aureus* (MRSA), but also against some Gram-negatives (*Moraxella* and *Neisseria*). The compound(s) identification will be our future aim, as it targets clinically relevant human pathogens. Interestingly, the antibacterial activity against some microbes (*S. aureus, S. pneumoniae, N. pharyngis*) raised in the TR1341ΔFIL2 mutant compared to the *wt*, but remained the same against all others (Supplementary Figure S2). The mutant also loses the ability of the wt (Figure 5 and Table 2) to induce the hemolysis in *S. aureus* and *P. aeruginosa*, but the capsule-suppressing effect on *S. pneumoniae* is retained.

### Human Cells-Targeted Activities of TR1341

**FIGURE 6:**
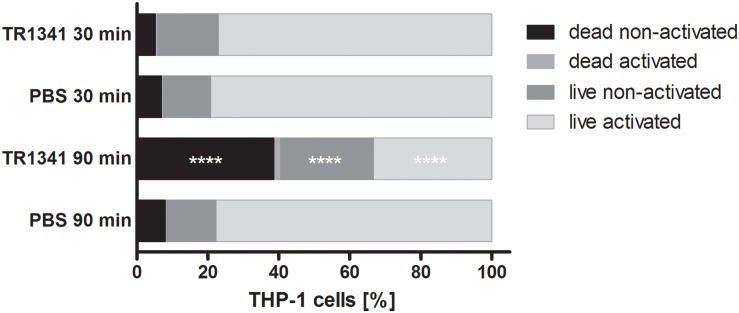
*In vivo* influence of TR1341 spores on human THP-1 monocytes after 30 and 90 min of coculture. Data are expressed as an average (n = 3) proportion of dead cells, dead but activated cells, neither activated nor dead cells and live activated cells. Control cells were cultivated in PBS only. ^****^P ≤ 0.0001 (two-way ANOVA).

The interactions of TR1341 with human cells was first assayed *in vivo*, using coculture of the strain spores with human monocyte cell line THP-1 (ratio 10: 1 = spores: macrophages). The viability and activation of THP-1 was assayed after 30 and 90 min of coculture as described in Section “Materials and Methods.” There was no effect neither in activation nor in survival of the THP-1 after 30 min coculture. But after 90 min, compared to PBS, a substantial drop in viability was obvious in the coculture with TR1341 spores – around 40% dead cells compared to about 8% in the controls. Also, number of viable, but not activated cells was significantly higher in the coculture: Above 26% compared to approx. 14% in the controls, suggesting the slight immunosuppressive effect of the germinating spores (Figure 6).

**FIGURE 7:**
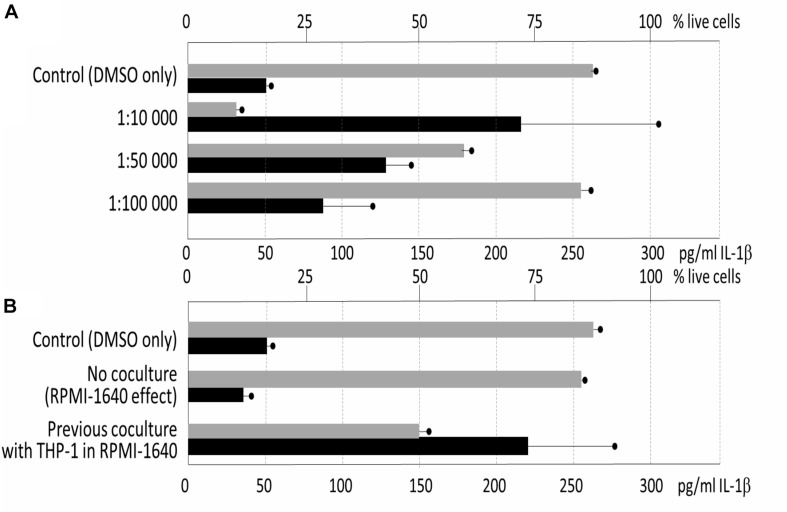
Influence of TR1341 extracts on THP-1 human macrophages. Viability of the cells indicated by gray rectangles (top axis scale), pro-inflammatory activation of the cells measured as IL-1β release indicated by black rectangles (bottom axis). In all the assays THP-1 were activated by LPS. **(A)** Standard fermentation in GYM media – effect of extract dilution (dilution indicated on the *Y* axis). Control = cells *+* LPS *+* DMSO. **(B)** Effect of preculture of TR1341 with THP-1 prior to fermentation on production of pro-inflammatory and cytotoxic compounds. Control as above, extracts diluted 1:5,000.

Next, the immunomodulatory effect of the metabolic extracts originated from the 3-days old stationary cultures was assayed for the cytotoxicity to THP-1. The experiment showed quite high level of cytotoxicity of the GYM media-derived culture with only 7% surviving cells in average in the 1: 10,000 dilution of the extract, and 64% in 1:50,000 dilution (compared to the control 88%, *p* = 0.001 and *p* = 0.0292, respectively). For the pro-inflammatory activity assays, measured as activation of IL-1β production in comparison with control, dilution of 1: 10,000 (*p* = 0.0362), 1: 50,000 (*p* = 0.0038) and 1: 100,000 (*p* = 0.0714) were used and the cells were simultaneously stimulated by LPS. The TR1341 extract caused a strong pro-inflammatory effect: the effect was concentration-dependent, dropping with the extract dilution, and statistically significant for the two lower dilutions of 1: 10,000 and 1: 50,000 (Figure 7A).

In the last experiment, we assayed whether the previous contact with THP-1 human cells may change secondary metabolome bioactivities in TR1341. The streptomycete early exponential phase mycelia were cocultured with THP-1 in the RPMI-1640 media for 6 hrs. The culture was used as seed culture in standard GYM fermentation. In a control, only RPMI-1640 media without THP-1 was used to assess the impact of the rich tissue culture media itself on the streptomycete metabolism. The extracts were prepared as in the previous experiment and assayed the same way. The cytotoxicity of the extracts was ten times lower then after the standard fermentation: 1: 10,000 diluted extract had no effect on the cell viability; 1: 5,000 diluted extracts reduced viability to 50% in the coculture experiment (*p* = 0.0019), but had no effect in the control (84% viable, *p* = 0.3794). The same dilution was used to assess the effect of the extracts on the IL-1β production. Despite the drop in the percentage of alive cells, the overall production of IL-1β was more than seven times higher (*p* = 0.0099). This means that TR1341 cells boost its pro-inflammatory effect upon the contact with human cells (Figure 7B).

**FIGURE 8:**
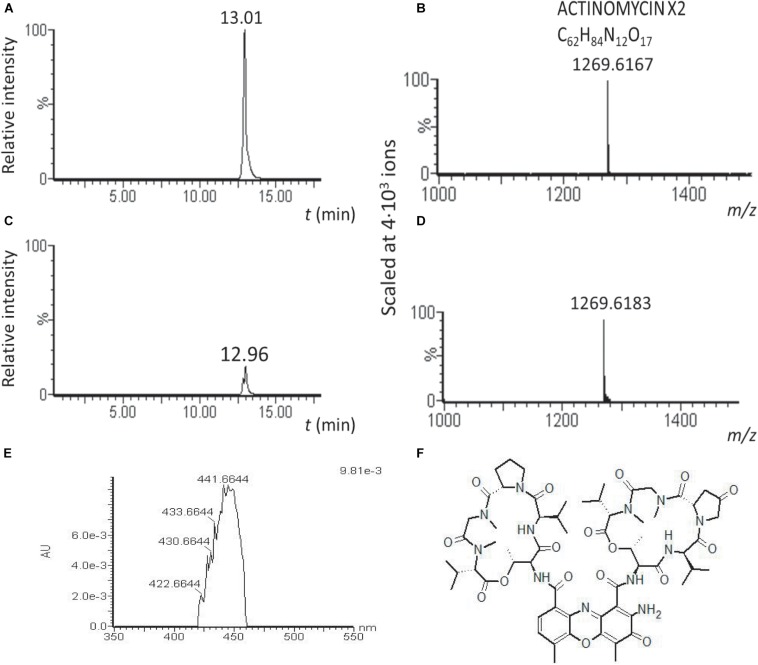
Actinomycin X2 production is substantially augmented in the TR1341ΔFIL2 mutant. LC-MS analysis of the acetone/ethylacetate extracts of *wt* and filipin mutant grown under standard conditions in GYM. Relative intensity of the actinomycin X2 signal in the TR1341ΔFIL2 mutant **(A)** is approximately 5 times higher than in the wt **(C)**. The molecular mass of the corresponding peaks is given in **(B,D)**. Absorption spectrum of the compound **(E)** corresponds to that of actinomycin X2 **(F)** (Furukawa et al., 1968).

We hypothesized that the high cytotoxicity of the TR1341 extracts originates in the production of the cytolytic polyenes. That is why the cytotoxicity of the TR1341ΔFIL2 was assessed, too. Surprisingly, it was even an order higher than in wt. As the genome of TR1341 contains a putative gene cluster encoding another highly cytotoxic compound, actinomycin (data not shown), we analyzed the *wt* and mutant extracts for the presence of any actinomycin derivative. Actinomycin X2 was, in concordance with the genetic data, detected in both extracts by LC-MS, but the peak relative intensity was approximately 5.4 times higher in the extract of the filipin mutant TR1341ΔFIL2 than in *wt* (Figure 8). Comparison of the peak areas in HPLC chromatogram confirms 3.8 times higher production of actinomycin X2 in the filipin mutant. This finding can well explain the high cytotoxicity of the mutant extract and may also cause stronger antibacterial activity of the mutant extract (Sharma and Manhas, 2019).

## Discussion

In this study, we attempted to assess what factors may predetermine a streptomycete strain to colonize human tissues successfully and, perhaps, to become pathogenic. Such data are completely missing, even in the case of the well-recognized pathogens, *S. somaliensis* and *S. sudanensis.* Many factors may play a role in human non-mycetoma opportunistic infections: the individual strain metabolic characteristics – character of hydrolytic enzymes and bioactive secondary metabolites, the host immune system status or superinfection by other microbes (Verma and Jha, 2019). The Supplementary Table S1 summarizes all clinical cases of non-mycetoma human infections described in the literature up to date.

Almost 30 years ago McNeil et al. (1990) postulated that streptomycetes should be considered as potential pathogens to humans, having a role in primary pulmonary and cutaneous infections in some patients. Additionally, they recognized streptomycetes as the fourth most common isolate from clinical specimens among aerobic actinomycetes (following *Nocardia asteroides*, *Actinomadura madurae*, and *N. brasiliensis*) and underlined the importance to be further studied by clinical microbiologists. By inspecting the previous studies, we found, that the human non-mycetoma streptomycete infections are not linked to particular species (Supplementary Table S1), even though the set of 28 streptomycete clinical isolates mainly from sputum and wounds in the Mc Neil’s work was biochemically characterized as a single species: *S. griseus*. However, we should take in account that all the early taxonomic data, based solely on biochemical characteristics of the strains, might be quite misleading. The available data indicate that diverse streptomycetes from the nearby environment can colonize human body and potentially cause infection. The *Streptomyces* sp. TR1341 strain was selected from the collection of human-associated streptomycete isolates for further characterization mostly due to its most complex bioactive compounds production and strong β-hemolytic features. According to phylogenetic analyses, the pneumonia-associated strain is closely related to *Streptomyces costaricanus* NBRC100773 by 16S rRNA taxonomy and belongs to the *S. murinus* group by autoMLST multi-locus taxonomy. None of the closely related strains has been linked to human infection, though some were reported as plant-associated (Figure 1 and Supplementary Figure S1). Strain showed typical streptomycete antibiotic susceptibility profile: it was susceptible to aminoglycosides, macrolides, and additionally to minocycline, vancomycin, and amoxicillin augmented by clavulanic acid and resistant to cephalosporins, quinolones, rifampicin, tetracycline, chloramphenicol and penicillins. However, it was resistant to trimethoprim-sulfamethoxazole (cotrimoxazole, COX), showing completely no inhibition zone. This is not so often among streptomycetes, being previously observed only in streptomycete isolate from lung nodule (Kapadia et al., 2007), in *Streptomyces cacaoi* isolated from scalp abscess (Pellegrini et al., 2012), and in 29% of clinical streptomycete isolates of the study of McNeil (McNeil et al., 1990). Interestingly, the patient had been treated with COX a few years prior to the strain isolation.

We hypothesize, that one of the characteristics significant for successful colonization of human tissue by certain streptomycetes is their secondary metabolites repertoire. Though taxonomically related, the family of *Streptomycetaceae* varies greatly in the secondary metabolites produced by individual species or even strains of a single species. About 2/3 of their linear genomes encodes adaptive functions: secondary metabolites, extracellular enzymes, resistance genes, etc., which can be easily exchanged in the bacterial communities by means of horizontal gene transfer (Egan et al., 2001; Barka et al., 2016). The secondary metabolites produced may not have only positive impacts on the putative hosts, as mentioned in connection to streptomycetes as a members of healthy human microbiota. Many strains produce cytotoxic or cytolytic compounds (Knopik-Skrocka and Bielawski, 2002), or compounds able to slow down or stop the host immune response, cell division, etc. (e.g., (Striz et al., 2008; Petrickova et al., 2014). People in regular contact with the soil dust (miners, farmers) as well as inhabitants of cities with high air pollution and water-damaged houses are the most exposed. Minor immune system disorders or the altered mucosa and skin fitness or superinfection may be the major factors of the successful colonization or even progression to disease.

Our data show that the predispositions of the TR1341 strain encompass the production of β-hemolytic compounds with fungicide activity (filipin and fungichromin) and the production of another highly cytotoxic compound, actinomycin X2. Both can have a deadly impact on any human cell (Knopik-Skrocka and Bielawski, 2002; Liu et al., 2016). We have proven that the only β-hemolytic factor of the TR1341 strain are polyene compounds of the filipin group. Moreover, the genomes of streptomycetes, including TR1341 and the type soil strain *S. coelicolor* A3(2) contain at least 4 putative genes homologous to protein hemolysins genes of human or animal pathogens. One of these is essential for the incomplete α-hemolysis of *S. coelicolor* (Table 1). The disruption of the filipin/fungichromin gene cluster in TR1341 destroyed the β-hemolytic features of the strain entirely, leaving just the α-hemolytic features. As expected, together with the loss of hemolysis, the mutant strain lost also its fungicidal activities. However, the antibacterial activity remained the same or was even stronger in the mutant than in the *wt* (the wild type is active against various Gram-positive and Gram-negative bacteria – see Table 2). To explain these unexpected results, we have compared the *wt* and mutant metabolic profile that documented substantially higher production of actinomycin X2 in the filipin mutant compared to the *wt*. As both metabolic pathways do not share the same precursors, this may be due to the better supply of energy (ATP) and cofactors (NADH, NADPH) for the actinomycin biosynthetic machinery after depletion of the competing filipin pathway. The actinomycin X2 also has antibacterial features that may explain the higher antibacterial activity of the mutant extract, too.

The hemolytic effect of the strain *in vivo* is stronger than that of its metabolic extract. The finding may have several reasons. It is well documented that the production of secondary metabolites is coregulated by global regulators with morphogenesis (Hou et al., 2018; Wang et al., 2018). Since the extractions were performed from liquid cultures where the streptomycete cells often do not undergo standard morphological differentiation, the overall secondary metabolism rates may be reduced. Next, filipin-type polyenes are prone to oxidations and subsequent loss of the bioactivity (Tingstad and Garrett, 1960), so they may be partially degraded during the extraction. The appearance of hemolytic zones caused by the culture and its extract is slightly different. Production of the putative α-hemolysin (SCO1782) homolog may contribute to the discoloration of the zone by hemoglobin degradation (Rajesh et al., 2013) *in vivo*.

Disruption of the polyene production stops the CAMP test-like induction of hemolysis in other pathogens (*S. aureus, P. aeruginosa*) by TR1341 in the coculture assays. We can expect that larger and clearer hemolytic zones around the β-hemolytic pathogens originate from the secretion of the cytolytic polyenes from the TR1341 wt mycelia into the agar media. On the other hand, polyene-less mutant still suppresses formation of the capsule in the *S. pneumoniae*. Of the possible mechanisms, which might be involved in the suppression, production of extracellular capsule-degrading enzymes or quorum quenching mechanisms should be considered.

Next, we assayed immunomodulatory impacts of the interaction of human THP-1 macrophage cells with germinating spores. The germinating spores can be considered as the first active cells that the human mucosa immune cells encounter and react to when they are inhaled. We have shown in *in vitro* experiments that human macrophages viability and ability to activate the proper response to an invader are significantly reduced by their contact with the TR1341 germinating spores. Additionally, we have monitored the effect of secondary metabolites produced by stationary TR1341 cultures on viability and pro-inflammatory response of THP-1. The extracts of the late culture showed high cytotoxicity that we first ascribed to the production of the cytolytic polyenes. However, the extract of the polyene non-producing mutant had the cytotoxic effects even 10 times higher, though it obviously lost hemolytic activities. Detailed comparison of LC-MS data of both strains revealed a substantial elevation of the actinomycin X2 production, another highly cytotoxic compound. Disregarding the cytotoxicity of the wt extract, in sub-cytotoxic dilutions, it stimulated the pro-inflammatory response of THP-1. The late, stationary cultures used for the metabolite extractions may mimic microcolonies putatively formed in the host tissue after successful colonization.

The data presented in this work cannot be considered as a proof of a direct link between the patient’s symptoms of relapsing atypical pneumonias and colonization of his lungs by *Streptomyces* sp. TR1341, though no other facultative or obligatory pathogens were found in the sputum. In order to assess the facultative pathogenicity of the strain, at least an animal (mouse) model of lung colonization/infection should be used, which we would like to do in the near future. However, we should mention that production of similar metabolites (antifungal and cytolytic polyenes, actinomycins and cytotoxic compounds) was often reported in actinomycetes associated with animals and plants: Filipins are produced by mutualistic actinomycetes of brown algae (Parrot et al., 2019) and water caltrop plant (Kim et al., 2012), larger candicidins by symbionts of ants (Haeder et al., 2009; Barke et al., 2010; McLean et al., 2016), sceliphrolactam by digger wasps symbionts (Oh et al., 2011; Poulsen et al., 2011) and linear micangymicins by actinomycetes associated with southern pine beetles (Oh et al., 2009). Similarly, actinomycins production is often associated with ants actinomycete symbionts (Schoenian et al., 2011; Boya et al., 2017), where also cancerostatic antimycins frequently appear (Poulsen et al., 2011; Seipke et al., 2011; McLean et al., 2016). This supports our hypothesis that human-associated streptomycetes may benefit from production of these compounds, too.

## Conclusion

This work documents several genetic adaptations that could help *Streptomyces* sp. TR1341 and perhaps also other human-associated streptomycetes to colonize the evolutionary new niche, the human tissue. First, it is a production of cytolytic or hemolytic polyenes, which allows the streptomycete not only to lyse human cells for nutrition, but also to conquer fungi. Second, it produces highly cytotoxic actinomycin X2. Third, the strain germinating spores can suppress the immune response of the human macrophages *in vitro*. The mechanism remains still unclear. Though the production of secondary metabolites is typical for stationary growth phases, small amounts of secondary metabolites can be detected already during the spore germination in *S. coelicolor* (Cihak et al., 2017). The observed suppression of THP-1 activation thus may originate from both direct interaction of TR1341 with macrophages and production of some bioactive compounds. Such activity would be quite beneficial for the streptomycete in the early phase of human lung tissue colonization as it could protect the spores and young mycelia from the attack of innate immune cells. Fourth, in contrast to the germinating spores, the stationary cultures of TR1341 produce secondary metabolites with pro-inflammatory and cytotoxic features. This feature becomes even stronger upon contact with human macrophages. Inflammatory processes are associated with destruction of the host tissue that may certainly provide the intruder with nutrients from the lysing host cells. Taking all the data together, we think that the crucial factors of pathogenicity in streptomycetes originate from their secondary metabolism. “Suitable” biosynthetic gene clusters may be easily spread by mechanisms of horizontal gene transfer in soil or other biotopes (Shintani et al., 2014; McDonald and Currie, 2017). This may explain why non-actinomycetoma streptomycete infections are reported even in taxonomically distant strains.

## Data Availability Statement

The datasets generated for this study can be found in the genomic NCBI: BioProject PRJNA558635, BioSample SAMN12496210, SRA SRR9903270 and WGS VSDL00000000.

## Author Contributions

AH made the genetic manipulations with the streptomycete strain, performed fermentations and cocultures with human cells, prepared the metabolic extracts, and performed cytotoxicity and immunomodulatory effects assays of the extracts. EC performed the phylogenetic analyses, analyzed the genomic data and made the hemolysis and growth-inhibitory activities assays *in vivo*. AC supervised the phylogenetic analyses and analyzed the data, performed the genome sequencing, and participated in the manuscript preparation. HL, PP, and JH assayed the human cells-targeted activities. HL designed and performed the *in vivo* cocultures and evaluated their effects. PP, JH, and AH designed and performed the experiments to assess immunomodulatory features of metabolic extracts and their cytotoxicity. JH and HL evaluated the human cells-related experiments data and participated in the manuscript preparation. MČ performed and evaluated the LC-MS experiments. VK made the strain taxonomic and morphologic characterization. JB was involved in the growth-inhibitory activities *in vivo* and in evaluation of the strain interactions with pathogenic microbes. MP made the overall design of extraction experiments and evaluated the data. KP planned the experiments, participated in the activity screenings, analyzed and interpreted the data, made the literature review on streptomycete-related human infections, and prepared the manuscript.

## Conflict of Interest

The authors declare that the research was conducted in the absence of any commercial or financial relationships that could be construed as a potential conflict of interest.

## Footnotes

1 https://www.asm.org/getattachment/7ec0de2b-bb16-4f6e-ba07-2aea25a43e76/protocol-2885.pdf

2 https://link.springer.com/article/10.1007%2Fs10482-016-0824-0#Sec3
