# Supplementary material for: A Human Lung-Associated Streptomyces sp. TR1341 Produces Various Secondary Metabolites Responsible for Virulence, Cytotoxicity and Modulation of Immune Response
Source: Front Microbiol. 2020 Jan 17;10:3028. doi: 10.3389/fmicb.2019.03028 (PMC6978741; doi:10.3389/fmicb.2019.03028)

Supplementary Figure S1. Multi-Locus sequence analysis based on 83 single copy marker gene calculated using autoMLST. *Actinospica acidiphila* NRRL B-24431 was used as outgroup and only bootstrap values higher than 50 are shown. Only strains with draft/complete genome were considered in this analysis. *Streptomyces* strains marked with dots were isolated from water (blue) or are plant associated (green), plant pathogens (purple), or human pathogens (red); all other strain were either isolated from soil or their isolation source could not be found in literature.

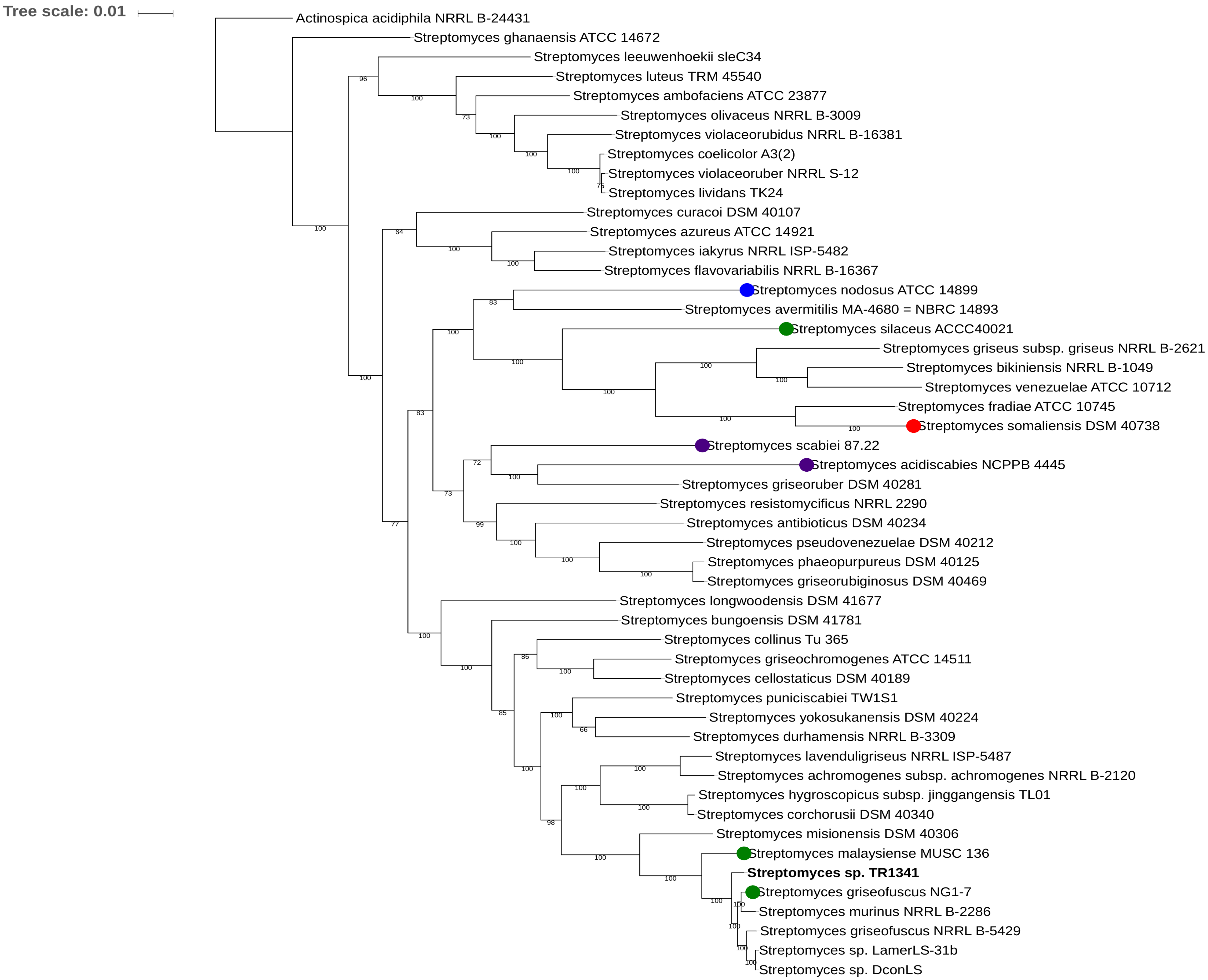

Supplement: Supplementary file 1 [file Image_1.pdf]
