# Supplementary material for: A Human Lung-Associated Streptomyces sp. TR1341 Produces Various Secondary Metabolites Responsible for Virulence, Cytotoxicity and Modulation of Immune Response
Source: Front Microbiol. 2020 Jan 17;10:3028. doi: 10.3389/fmicb.2019.03028 (PMC6978741; doi:10.3389/fmicb.2019.03028)

Supplementary Figure S2. Antibiotic activities of the *S. sp.* TR1341 wt and TR1341ΔFIL2 mutant. The mutant extract makes bigger growth-inhibiting zones in some bacteria (*Staphylococcus aureus*, *Neisseria lactamica*, *Streptococcus pneumoniae* and *Klebsiella pneumoniae*), in others the zones do not change in the filipin mutant (*Bacillus subtilis*, *Moraxella catarrhalis*). Representative results shown for *Streptococcus pneumoniae* **(A)** and *Moraxella catarrhalis* **(B)**. Ten µl of the extract were applied to TLC, the chromatographic plate was air-dried and printed onto the surface of blood agar plate inoculated by standardized suspension of the assayed organism. The plates were then cultured overnight at 37 oC. In general, the size of the zone for a particular organism-compound pair sums up several factors: diffusibility and stability of the antibiotic, the organism sensitivity to the compound and the organism growth rate.

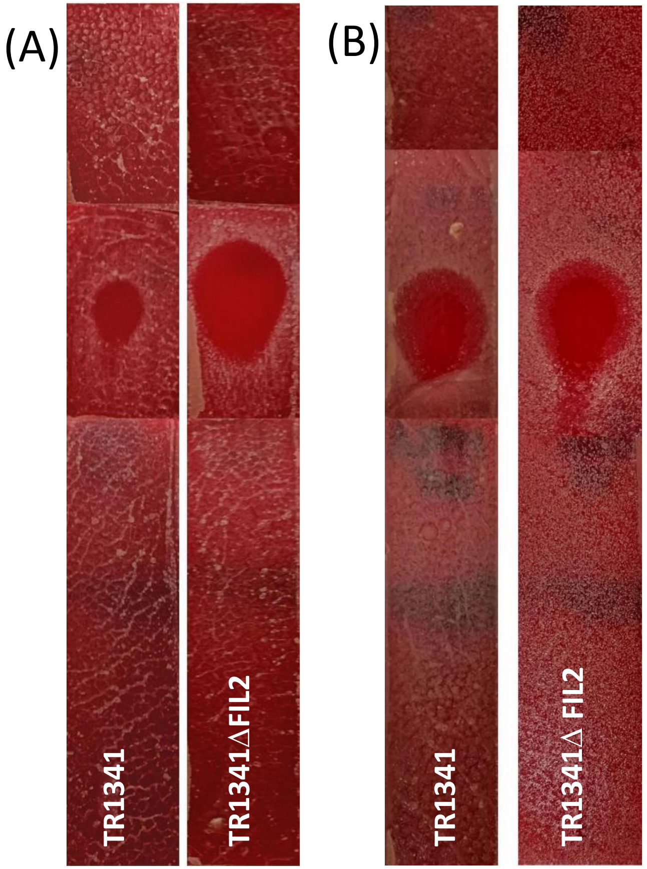

Supplement: Supplementary file 2 [file Image_2.pdf]
