# Supplementary material for: A Human Lung-Associated Streptomyces sp. TR1341 Produces Various Secondary Metabolites Responsible for Virulence, Cytotoxicity and Modulation of Immune Response
Source: Front Microbiol. 2020 Jan 17;10:3028. doi: 10.3389/fmicb.2019.03028 (PMC6978741; doi:10.3389/fmicb.2019.03028)
Supplement: Supplementary file 3 [file Table_1.pdf]

Supplementary Table 1. Review of streptomycete-related human infections. M/F-x stands for the gender and age of the patient, when known (ND – not defined). Underlying diseases and other clinical info given in the Notes column. In the older works (approx. before the year of 2000), the taxonomy of the strains, based solely on their biochemical characteristics, may not be reliable.

Clinical manifestation		Strain isolated	Notes	Reference
**Lung infections**				
Lung nodule	1	*S. maritimus/olivaceus*	F-21, AML with neutropenia	(Kapadia et al., 2007)
	2	*S. albus*	F-23, systemic lupus erythematosus	(Kapadia et al., 2007)
	3	*S. sp.*	F-69	(Canoui et al., 2019)
Hypersensitivity pneumonitis	1	*S. sp.*	M-18, Burkitt lymphoma	(Kapadia et al., 2007)
	2	*S. sp.*	ND	(Tiotiu et al., 2013)
Fulminant pneumonitis	1	*S. thermoviolaceous*	M-23, immunodeficient, coinfection with fungi, mulch contact indicated	(Siddiqui et al., 2007)
	2	*S. sp.*	M-18, immunodeficient, coinfection with fungi, mulch contact indicated	(Siddiqui et al., 2007)
	3	*S. sp.*	M-10, immunodeficient, coinfection with fungi, mulch contact indicated	(Siddiqui et al., 2007)
Pneumonia/ bronchopneumonia	1	*S. pelletieri*	Neonate infection, the same organism in the mother vaginal discharge	(Werder and Sonnabend, 1973)
	2	*S. sp.*	M-43, HIV infection	(Dunne et al., 1998)
	3	*S. rectiverticillatus/N. aureoverticillis*	M-85, with gastrectomy	(Matsunaga et al., 2009)
	4	*S. lanatus*	F-52	(Kofteridis et al., 2007)
	5	*S. sp.*	M-57, sarcoidosis, splenectomy	(Riviere et al., 2012)
	6	*S. griseus*	ND	(Gugnani et al., 1993)
	7	*S. cinereoruber*	M-68, brick-layer, heavy smoker, coinfection with *Haemophilus influenzae*	(Manteca et al., 2008)
	8	*S. sp.*	M-30, AIDS, nodular form	(Caron et al., 1992)
	9	*S. sp.*	M-35, AIDS, with monoarthritis	(Ahmed et al., 1996)
	10	*S. sp.*	F-61, previous lung cancer, postobstructive necrotizing pneumonia	(Yacoub et al., 2014)
	11	*S. griseus*	10 cases reported to Centers of Disease Control (CDC) between Nov 1985 – Feb 1988	(McNeil et al., 1990)
Chronic bronchitis		*S. sp.*	ND, repeated isolation of the strain within 1 year of chronic bronchitis	(Scharfen et al., 2000)
Farmer's Lung Disease		*S. thermohygroscopicus*	ND	(Zhu, 1989)
Hydropneumothorax		*S. sp.*	M-24, Cushing syndrome	(Ataiekhorasgani et al., 2014)
Cystic fibrosis		*S. violaceorectus*	ND	www.ncbi.nlm.nih.gov/ biosample/3252885
**Bacteremia, blood infections**	1	*S. sp.*	ND-50, pneumonia	(Kohn et al., 1951)
	2	*S. sp.*	M-56, prosthetic valvae, endocarditis	(Mossad et al., 1995)
	3	*S. sp.*	M-49, breast cancer, catheter-related	(Carey et al., 2001)
	4	*S. bikinensis*	F-14, osteosarcoma	(Moss et al., 2003)
	5	*S. somaliensis*	ND; Intraspinal mycetoma	(Arbab et al., 1997)
	6	*S. thermovulgaris*	F-81, Crohn's Disease	(Ekkelenkamp et al., 2004)
	7	*S. nobilis/parvulus*	M-71, metastatic cholangiocarcinoma	(Kapadia et al., 2007)
	8	*S. humidus/pilosus*	F-51, metastatic breast cancer	(Kapadia et al., 2007)
	9	*S. indigoferus/herbaricolor*	M-25, Ewing sarcoma, neutropenia	(Kapadia et al., 2007)
	10	*S. atratus*	M-77, community-acquired pneumonia	(Ariza-Prota et al., 2015)
	11	*S. sp.*	M-66, prosthetic valvae	(Shehatha and Taha, 2017)
	12	*S. sp.*	F-29 with actinomycetoma	(Joseph et al., 2010)
	13	*S. sp.*	ND, septic thrombosis	(Ghanem et al., 2007)

	14	*S. cacaoi* ssp. *cacaoi*	M-74, dental abscess-originated	(Kazarian and Kauffman, 2014)
	15	*S. griseus*	7 cases reported to CDC between Nov 1985 – Feb 1988	(McNeil et al., 1990)
**Brain abscess**	1	*S. griseus*	F-53, with sinusitis, meningitis	(Clarke et al., 1964)
	2	*S. caelestis*	M-19, skull-penetrating injury with a rake	(Rose et al., 2008)
	3	*S. griseus*	2 cases reported to CDC between Nov 1985 – Feb 1988	(McNeil et al., 1990)
**Skin infection**				
Pitted keratolysis			Multiple cases, Tatami-associated, healthy young athletes, other actinomycetes involved too, often misdiagnosed	(Balic et al., 2018)
Cutaneous abscesses			M-19, immunodeficient	(Zayet et al., 2019)
**Other**				
Chronic pericarditis		*S. sp.*	ND-25	(Shanley et al., 1979)
Peritonitis	1	*S. somaliensis*	ND-58	(Gruet et al., 1970)
	2	*S. viridis*	M-53, chronic alcoholic	(Datta et al., 2012)
Lymphadenitis		*S. sp.*	ND-26, AIDS, IV drug abuse	(Holtz et al., 1985)
Mammary implant infection		*S. sp.* (close to *albus*)	F-48, mastectomy, carcinoma	(Manteca et al., 2009)
Scalp abscess		*S. cacaoi ssp. cacaoi*	F-31, post-injury with a log	(Kazarian and Kauffman, 2014)
Keratitis		*S. thermocarboxydus*	M-50	(Kawakami et al., 2014)
Wound infection		*S. griseus*	9 cases reported to CDC between Nov 1985 – Feb 1988	(McNeil et al., 1990)

References:

Ahmed, A.J., Ali, S.T., Weinbaum, D., and Goldberg, E. (1996). *Streptomyces* infection in AIDS presenting with pneumonia and monoarthritis. Inf. Dis. Clin. Pract. 5, 207-208.

Arbab, M.A., el Hag, I.A., Abdul Gadir, A.F., and Siddik, H.e.-R. (1997). Intraspinal mycetoma: report of two cases. Am. J. Trop. Med. Hyg. 56(1), 27-29. doi: 10.4269/ajtmh.1997.56.27.

Ariza-Prota, M.A., Pando-Sandoval, A., Fole-Vazquez, D., Garcia-Clemente, M., Budino, T., and Casan, P. (2015). Community-acquired bacteremic *Streptomyces atratus* pneumonia in animmunocompetent adult: a case report. J. Med. Case Rep. 9, 262. doi: 10.1186/s13256-015-0753-y.

Ataiekhorasgani, M., Jafaripozve, N., and Zaerin, O. (2014*). Streptomyces* infection in Cushing syndrome: A case report and literature review. Adv. Biomed. Res. 3, 26. doi: 10.4103/2277-9175.124672.

Balic, A., Bukvic Mokos, Z., Marinovic, B., and Ledic Drvar, D. (2018). Tatami Mats: A Source of Pitted Keratolysis in a Martial Arts Athlete? Acta Dermatovenerol. Croat. 26(1), 68-70.

Canoui, E., Ingen-Housz-Oro, S., Ortonne, N., Lebeaux, D., Rodriguez-Nava, V., Godeau, B., et al. (2019). Hemophagocytic lymphohistiocytosis with granulomatosis and diffuse T-cell infiltration associated with disseminated nocardiosis and pulmonary infection due to *Streptomyces* spp. Rev. Med. Interne. doi: 10.1016/j.revmed.2019.04.013.

Carey, J., Motyl, M., and Perlman, D.C. (2001). Catheter-related bacteremia due to *Streptomyces* in a patient receiving holistic infusions. Emerg. Infect. Dis. 7(6), 1043-1045. doi: 10.3201/eid0706.010624.

Caron, F., Borsa-Lebas, F., Boiron, P., Vasseur, E., Hennequin, C., Nouvellon, M., et al. (1992). *Streptomyces* sp. as a cause of nodular pneumonia in an HIV infected patient. Med. Micriobiol. Lett. 1, 297-303.

Clarke, P.R., Warnock, G.B., Blowers, R., and Wilkinson, M. (1964). Brain Abscess Due to *Streptomyces griseus*. J. Neurol. Neurosurg. Psychiatry 27, 553-555. doi: 10.1136/jnnp.27.6.553.

Datta, P., Arora, S., Jain, R., Chander, J., and van de Sande, W. (2012). Secondary peritonitis caused by *Streptomyces viridis*. J Clin. Microbiol. 50(5), 1813-1814. doi: 10.1128/JCM.06045-11.

Dunne, E.F., Burman, W.J., and Wilson, M.L. (1998). *Streptomyces* pneumonia in a patient with human immunodeficiency virus infection: case report and review of the literature on invasive streptomyces infections. Clin. Infect. Dis. 27(1), 93-96. doi: 10.1086/514612.

Ekkelenkamp, M.B., de Jong, W., Hustinx, W., and Thijsen, S. (2004). *Streptomyces thermovulgaris* bacteremia in Crohn's disease patient. Emerg. Infect. Dis. 10(10), 1883-1885. doi: 10.3201/eid1010.040300.

Ghanem, G., Adachi, J., Han, X.Y., and Raad, I. (2007). Central venous catheter-related *Streptomyces* septic thrombosis. Infect. Control Hosp. Epidemiol. 28(5), 599-601. doi: 10.1086/513619.

Gruet, M., Maydat, L., and Ferro, R. (1970). Peritonitis caused by *Streptomyces somaliensis*. Bull. Soc. Med. Afr. Noire. Lang. Fr. 15(4), 609-610.

Gugnani, A.C., Unaogu, I.C., and Emeruwa, C.N. (1993). Pulmonary infection due to *Streptomyces griseus*. J. Commun. Dis. 25(1), 38-40.

Holtz, H.A., Lavery, D.P., and Kapila, R. (1985). *Actinomycetales* infection in the acquired immunodeficiency syndrome. Ann. Intern. Med. 102(2), 203-205.

Joseph, N.M., Harish, B.N., Sistla, S., Thappa, D.M., and Parija, S.C. (2010). *Streptomyces* bacteremia in a patient with actinomycotic mycetoma. J. Infect. Dev. Ctries 4(4), 249-252.

Kapadia, M., Rolston, K.V.I., and Han, X.Y. (2007). Invasive *Streptomyces* infections - Six cases and literature review. Am. J. Clin. Pathol. 127(4), 619-624. doi: 10.1309/QJEBXP0BCGR54L15.

Kawakami, H., Inuzuka, H., Mochizuki, K., Muto, T., Ohkusu, K., Yaguchi, T., et al. (2014). Case of keratitis caused by *Streptomyces thermocarboxydus*. J. Infect. Chemother. 20(1), 57-60. doi: 10.1016/j.jiac.2013.11.001.

Kazarian, E.R., and Kauffman, C.A. (2014). *Streptomyces* Bacteremia: Case Report and Review of the Literature. Inf. Dis. Clin. Practice 22(4), 194-197. doi: 10.1097/ipc.0000000000000124.

Kofteridis, D.P., Maraki, S., Scoulica, E., Tsioutis, C., Maltezakis, G., and Gikas, A. (2007). *Streptomyces* pneumonia in an immunocompetent patient: a case report and literature review. Diag. Microbiol. Inf. Dis. 59(4), 459-462.

Kohn, P.M., Tager, M., Siegel, M.L., and Ashe, R. (1951). Aerobic actinomyces septicemia report of a case. N. Engl. J. Med. 245(17), 640-644. doi: 10.1056/NEJM195110252451703.

Manteca, A., Pelaez, A.I., del Mar Garcia-Suarez, M., Hidalgo, E., del Busto, B., and Mendez, F.J. (2008). A rare case of lung coinfection by *Streptomyces cinereoruber* and *Haemophilus influenzae* in a patient with severe chronic obstructive pulmonary disease: characterization at species level using molecular techniques. Diagn. Microbiol. Infect. Dis. 60(3), 307-311. doi: 10.1016/j.diagmicrobio.2007.10.009.

Manteca, A., Pelaez, A.I., del Mar Garcia-Suarez, M., Hidalgo, E., Lopez, S., and Mendez, F.J. (2009). A rare case of silicone mammary implant infection by *Streptomyces* spp. in a patient with breast reconstruction after mastectomy: taxonomic characterization using molecular techniques. Diagn. Microbiol. Infect. Dis. 63(4), 390-393. doi: 10.1016/j.diagmicrobio.2008.12.010.

Matsunaga, K., Nagata, N., Wakamatsu, K., Iwata, Y., Kumazoe, H., Komori, M., et al. (2009). A case of *Streptomyces* pneumonia. Nihon Kokyuki Gakkai Zasshi 47(7), 569-574.

McNeil, M.M., Brown, J.M., Jarvis, W.R., and Ajello, L. (1990). Comparison of Species Distribution and Antimicrobial Susceptibility of Aerobic Actinomycetes from Clinical Specimens. Rev. Infect. Dis. 12(5), 778-783.

Moss, W.J., Sager, J.A., Dick, J.D., and Ruff, A. (2003). *Streptomyces bikiniensis* bacteremia. Emerg. Infect. Dis. 9(2), 273-274.

Mossad, S.B., Tomford, J.W., Stewart, R., Ratliff, N.B., and Hall, G.S. (1995). Case report of *Streptomyces endocarditis* of a prosthetic aortic valve. J. Clin. Microbiol. 33(12), 3335-3337.

Riviere, E., Neau, D., Roux, X., Lippa, N., Roger-Schmeltz, J., Mercie, P., et al. (2012). Pulmonary *Streptomyces* infection in patient with sarcoidosis, France, 2012. Emerg. Infect. Dis. 18(11), 1907-1909. doi: 10.3201/eid1811.120797.

Rose, C.E., 3rd, Brown, J.M., and Fisher, J.F. (2008). Brain abscess caused *by Streptomyces* infection following penetration trauma: case report and results of susceptibility analysis of 92 isolates of *Streptomyces* species submitted to the CDC from 2000 to 2004. J. Clin. Microbiol. 46(2), 821-823. doi: 10.1128/JCM.01132-07.

Scharfen, J., Bunček, M., Ježek, P., Urbášková, P., Fridrichová, M., Krištůfek, V., et al. (2000). Filamentous „contaminants“ in the mycobacteriology laboratory; their culture, identification and clinical significance. Klin. Mikrobiol. Inf. Lek. 16(1):48-57.

Shanley, J.D., Snyder, K., and Child, J.S. (1979). Chronic pericarditis due to a *Streptomyces* species. Am. J. Clin. Pathol. 72(1), 107-110. doi: 10.1093/ajcp/72.1.107.

Shehatha, J.S., and Taha, A.Y. (2017). Early-onset *Streptomyces* endocarditis in a prosthetic aortic valve. Asian Cardiovasc. Thorac. Ann. 25(2), 137-139. doi: 10.1177/0218492315609458.

Siddiqui, S., Anderson, V.L., Hilligoss, D.M., Abinun, M., Kuijpers, T.W., Masur, H., et al. (2007). Fulminant mulch pneumonitis: an emergency presentation of chronic granulomatous disease. Clin. Infect. Dis. 45(6), 673-681. doi: 10.1086/520985.

Tiotiu, A., Metz-Favre, C., Reboux, G., Kessler, R., and de Blay, F. (2013). Hypersensitivity pneumonitis related to *Penicillium chrysogenum* and mesophilic *Streptomyces*: the usefulness of the Medical Indoor Environment Councelor (MIEC)]. Rev. Pneumol. Clin. 69(5), 278-282. doi: 10.1016/j.pneumo.2013.02.008.

Werder, E.A., and Sonnabend, W. (1973). Neonatal infection with *Streptomyces pelletieri*. Am. J. Dis. Child. 125(3), 439-441.

Yacoub, A.T., Velez, A.P., Khwaja, S.I., Sandin, R.L., and Greene, J. (2014). *Streptomyces* Pneumonia in an Immunocompromised Patient: A Case Report and a Review of Literature. Inf. Dis. Clin. Pract. 22(4), e113-e115. doi: 10.1097/IPC.0000000000000172.

Zayet, S., Berriche, A., Battikh, H., Zribi, M., Fendri, C., and Benaissa, H.T. (2019). Cutaneous abscesses with *Streptomyces rectiverticillatus* during an interleukin 12 deficiency. Presse Med. 48(1 Pt 1), 85-87. doi: 10.1016/j.lpm.2018.09.017.

Zhu, J.Y. (1989). A new pathogen of farmer's lung disease-*Streptomyces thermohygroscopicus* and its antigen analysis related to diagnosis. Zhonghua Yi Xue Za Zhi 69(12), 687-689, 648.
