# Supplementary material for: A Human Lung-Associated Streptomyces sp. TR1341 Produces Various Secondary Metabolites Responsible for Virulence, Cytotoxicity and Modulation of Immune Response
Source: Front Microbiol. 2020 Jan 17;10:3028. doi: 10.3389/fmicb.2019.03028 (PMC6978741; doi:10.3389/fmicb.2019.03028)
Supplement: Supplementary file 4 [file Table_2.pdf]

Supplementary Table S2. *Streptomyces* strains related to *Streptomyces* sp. TR1341 and used for the construction of the 16S rRNA gene ML tree. Non-soil origin indicated in the same color code as in Fig. 1. Pathogenic organisms shaded grey.

	Closest relative	bp	%	Accession No.	Isolation source
**1**	*S. ambofaciens* ATCC 23877	1583/1630	97	CP012382.1	Soil, France: Picardie
**2**	*S. atriruber* NRRL B-24165	1587/1630	97	NZ_LIPN01000196.1	Placenta, Equine, USA
**3**	*S. avermitilis* MA-4680 (NBRC 14893)	1588/1633	97	BA000030.4	Soil, Japan
**4**	*S. coelicolor* A3(2)	1495/1537	97	NC_003888.3	Soil
**5**	*S. collinus* Tu 365	1583/1628	97	CP006259.1	Soil
**6**	*S. costaricanus* NBRC 100773	1480/1480	100	NR_041414.1	Nematode-suppressive soil , Costa Rica
**7**	*S. daghestanicus* NRRL B-5418	1441/1484	97	NR_043816.1	Soil
**8**	*S. fuscichromogenes* m16	1395/1421	98.2	KC771428	Soil, tropical rain forest of Xishuangbanna, China
**9**	*S. graminearus* NBRC 15420	1446/1448	100	AB184667	-
**10**	*S. griseochromogenes* ATCC 14511	1587/1626	98	CP016279.1	-
**11**	*S. griseofuscus* KACC 20083	1487/1491	99	AY207605.1	-
**12**	*S. griseofuscus* NBRC 12870	1446/1447	100	AB184206	-
**13**	*S. griseofuscus* 199	1535/1535	100	NZ_PDES01000029.1	Endophytic of *Paullinia cupana* (Mart) Ducke, Brazil
**14**	*S. griseofuscus* NG1-7	1622/1626	99	NZ_MTKC01000003.1	Rotten tree, South Korea
**15**	*S. griseoviridis* F1-27	1592/1627	98	CP034687.1	USA
**16**	*S. griseus* subsp. *griseus* NBRC 13350	1572/1631	96	AP009493.1	Soil
**17**	*S. hygroscopicus* subsp. *jinggangensis* TL01	1580/1626	97	CP003720.1	-
**18**	*S. koyangensis* VK-A60T	1587/1626	98	CP031742.1	Soil, South Korea: Koyang
**19**	*S. lanatus* NBRC 12787	1420/1446	98.2	AB184845	Soil
**20**	*S. leeuwenhoekii* sleC34	1595/1636	97	LN831790.1	Salar de Atacama, Atacama Desert
**21**	*S. lividans* TK24	1494/1537	97	CP009124.1	-
**22**	*S. malaysiense* MUSC 136	1604/1628	99	NZ_LBDA02000093.1	Mangrove, Malaysia
**23**	*S. misionensis* DSM 40306	1605/1628	99	NZ_FNTD01000004.1	-
**24**	*S. murinus* NBRC 12799	1446/1446	100	AB184155	Soil, Germany
**25**	*S. murinus* NRRL B-2286	1623/1628	99	NZ_MUNF01000366.1	Soil, Germany
**26**	*S. nigra 452*	1588/1631	97	CP029043.1	mangrove soil, China
**27**	*S. nodosus ATCC 14899*	1584/1634	97	CP009313.1	Orinoco river basin
**28**	*S. padanus ATCC 25646*	1519/1519	100	AF455813.1	-
**29**	*S. phaeogriseichromatogenes NRRL 2834*	1334/1340	99.6	AJ391813	Soil, Sri Lanka
**30**	*S. phaeoluteichromatogenes NRRL 5799*	1408/1428	98.6	AJ391814	Rothamsted experiment station
**31**	*S. pseudogriseolus NRRL B-3288*	1584/1625	97	NZ_MUNG01000290.1	Soil, Japan
**32**	*S. puniciscabiei TW1S1*	1586/1626	98	CP017248.1	Soil, South Korea
**33**	*S. qaidamensis S10*	1589/1640	97	CP015098.1	Soil, China
**34**	*S. silaceus NRRL B-24166*	1586/1625	98	NZ_LIRJ01000287.1	Placenta, Equine, USA
**35**	*S. sp. DconLS*	1625/1628	99	NZ_FMDI01000408.1	-
**36**	*S. sp. endophyte_N2*	1627/1628	99	CP028719.1	*Arabidopsis thaliana*, United Kingdom: Norwich laboratory
**37**	*S. sp. ETH9427*	1590/1631	97	CP029624.1	Soil, Switzerland
**38**	*S. sp. F-1*	1605/1628	99	NZ_FKJI03000002.1	Soil
**39**	*S. sp. Lamer LS-31b*	1535/1535	100	NZ_FMDH01000239.1	-
**40**	*S. sp. MD1*	1518/1519	99	HM641908.1	Golf course soil, USA: Florida
**41**	*S. sp. P127*	1517/1517	100	EU797798.1	Soil sample collected from farmland growing vegetables, China
**42**	*S. sp. SAT1*	1580/1628	97	CP015849.1	*Adenophora trachelioides* Maxim, China
**43**	*S. sp. Z022*	1600/1629	98	CP033073.1	Grass soil, China: Dangxiong
**44**	*S. violaceoruber S21*	1571/1631	96	CP020570.1	Seabed sludge, China

**45**	*S. acidiscabies ATCC 49003*	1485/1539	96	AB026220.1	Potato scab, USA
**46**	*S. scabiei E-1*	1566/1635	96	AB026216.1	-
**47**	*S. scabiei 87.22*	1549/1629	95	FN554889.1	-
**48**	*S. somaliensis SL-3*	1483/1521	98	KF973254.1	*Morus alba*, China
**49**	*S. somaliensis DSM 40738*	1445/1486	97	NR_025292.1	Mycetoma on foot of 20-year old male, Somalia
**50**	*S. sudanensis SD 504*	1469/1522	97	NR_116037.1	Mycetoma patient
**51**	*S. sudanensis A29*	1436/1488	97	EF540897.1	Mycetoma patient
