# Supplementary material for: A Human Lung-Associated Streptomyces sp. TR1341 Produces Various Secondary Metabolites Responsible for Virulence, Cytotoxicity and Modulation of Immune Response
Source: Front Microbiol. 2020 Jan 17;10:3028. doi: 10.3389/fmicb.2019.03028 (PMC6978741; doi:10.3389/fmicb.2019.03028)
Supplement: Supplementary file 5 [file Table_3.pdf]

Supplementary Table S3. List of house-keeping genes used in the AutoMLST analysis

TIGRFAMs Accession No.	Name	Mainrole category	Function
**TIGR00133**	gatB	Protein synthesis	aspartyl/glutamyl-tRNA(Asn/Gln) amidotransferase, B subunit
**TIGR01798**	cit_synth_I	Energy metabolism	citrate (Si)-synthase
**TIGR00138**	rsmG_gidB	Protein synthesis	16S rRNA (guanine(527)-N(7))-methyltransferase RsmG
**TIGR01816**	sdhA_forward	Energy metabolism	succinate dehydrogenase, flavoprotein subunit
**TIGR01520**	FruBisAldo_II_A	Energy metabolism	fructose-bisphosphate aldolase, class II
**TIGR00036**	dapB	Amino acid biosynthesis	4-hydroxy-tetrahydrodipicolinate reductase
**TIGR01529**	argR_whole	Regulatory functions	arginine repressor
**TIGR00763**	lon	Protein fate	endopeptidase La
**TIGR02692**	tRNA_CCA_actino	Protein synthesis	CCA tRNA nucleotidyltransferase
**TIGR00338**	serB	Amino acid biosynthesis	phosphoserine phosphatase SerB
**TIGR00963**	secA	Protein fate	preprotein translocase, SecA subunit
**TIGR01127**	ilvA_1Cterm	Amino acid biosynthesis	threonine ammonia-lyase
**TIGR00459**	aspS_bact	Protein synthesis	aspartate--tRNA ligase
**TIGR00755**	ksgA	Protein synthesis	ribosomal RNA small subunit methyltransferase A
**TIGR00754**	bfr	Transport and binding proteins	bacterioferritin
**TIGR02504**	NrdJ_Z	Purines, pyrimidines, nucleosides, and nucleotides	ribonucleoside-diphosphate reductase, adenosylcobalamin-dependent
**TIGR00521**	coaBC_dfp	Biosynthesis of cofactors, prosthetic groups, and carriers	phosphopantothenoylcysteine decarboxylase / phosphopantothenate--cysteine ligase
**TIGR03594**	GTPase_EngA	Protein synthesis	ribosome-associated GTPase EngA
**TIGR00088**	trmD	Protein synthesis	tRNA (guanine(37)-N(1))-methyltransferase
**TIGR00086**	smpB	Protein synthesis	SsrA-binding protein
**TIGR00615**	recR	DNA metabolism	recombination protein RecR
**TIGR01039**	atpD	Energy metabolism	ATP synthase F1, beta subunit
**TIGR01134**	purF	Purines, pyrimidines, nucleosides, and nucleotides	amidophosphoribosyltransferase
**TIGR01137**	cysta_beta	Amino acid biosynthesis	cystathionine beta-synthase
**TIGR00690**	rpoZ	Transcription	DNA-directed RNA polymerase, omega subunit
**TIGR01032**	rplT_bact	Protein synthesis	ribosomal protein bL20
**TIGR00019**	prfA	Protein synthesis	peptide chain release factor 1
**TIGR01978**	sufC	Biosynthesis of cofactors, prosthetic groups, and carriers	FeS assembly ATPase SufC
**TIGR01225**	hutH	Energy metabolism	histidine ammonia-lyase
**TIGR00554**	panK_bact	Biosynthesis of cofactors, prosthetic groups, and carriers	pantothenate kinase
**TIGR02127**	pyrF_sub2	Purines, pyrimidines, nucleosides, and nucleotides	orotidine 5'-phosphate decarboxylase
**TIGR01302**	IMP_dehydrog	Purines, pyrimidines, nucleosides, and nucleotides	inosine-5'-monophosphate dehydrogenase
**TIGR03800**	PLP_synth_Pdx2	Biosynthesis of cofactors, prosthetic groups, and carriers	pyridoxal 5'-phosphate synthase, glutaminase subunit Pdx2
**TIGR01029**	rpsG_bact	Protein synthesis	ribosomal protein uS7
**TIGR01024**	rplS_bact	Protein synthesis	ribosomal protein bL19

**TIGR00382**	clpX	Protein fate	ATP-dependent Clp protease, ATP-binding subunit ClpX
**TIGR00065**	ftsZ	Cellular processes	cell division protein FtsZ
**TIGR00064**	ftsY	Protein fate	signal recognition particle-docking protein FtsY
**TIGR00060**	L18_bact	Protein synthesis	ribosomal protein uL18
**TIGR00062**	L27	Protein synthesis	ribosomal protein bL27
**TIGR01394**	TypA_BipA	Regulatory functions	GTP-binding protein TypA/BipA
**TIGR00639**	PurN	Purines, pyrimidines, nucleosides, and nucleotides	phosphoribosylglycinamide formyltransferase
**TIGR00436**	era	Protein synthesis	GTP-binding protein Era
**TIGR00302**	TIGR00302	Purines, pyrimidines, nucleosides, and nucleotides	phosphoribosylformylglycinamidine synthase, purS protein
**TIGR00431**	TruB	Protein synthesis	tRNA pseudouridine(55) synthase
**TIGR01980**	sufB	Biosynthesis of cofactors, prosthetic groups, and carriers	FeS assembly protein SufB
**TIGR00263**	trpB	Amino acid biosynthesis	tryptophan synthase, beta subunit
**TIGR01011**	rpsB_bact	Protein synthesis	ribosomal protein uS2
**TIGR01855**	IMP_synth_hisH	Amino acid biosynthesis	imidazole glycerol phosphate synthase, glutamine amidotransferase subunit
**TIGR02067**	his_9_HisN	Amino acid biosynthesis	histidinol-phosphatase
**TIGR01083**	nth	DNA metabolism	endonuclease III
**TIGR00577**	fpg	DNA metabolism	DNA-formamidopyrimidine glycosylase
**TIGR03705**	poly_P_kin	Central intermediary metabolism	polyphosphate kinase 1
**TIGR01169**	rplA_bact	Protein synthesis	ribosomal protein uL1
**TIGR00482**	TIGR00482	Biosynthesis of cofactors, prosthetic groups, and carriers	nicotinate (nicotinamide) nucleotide adenylyltransferase
**TIGR01994**	SUF_scaf_2	Biosynthesis of cofactors, prosthetic groups, and carriers	SUF system FeS assembly protein, NifU family
**TIGR01009**	rpsC_bact	Protein synthesis	ribosomal protein uS3
**TIGR00168**	infC	Protein synthesis	translation initiation factor IF-3
**TIGR00042**	TIGR00042	DNA metabolism	non-canonical purine NTP pyrophosphatase, RdgB/HAM1 family
**TIGR02970**	succ_dehyd_cytB	Energy metabolism	succinate dehydrogenase, cytochrome b556 subunit
**TIGR00048**	rRNA_mod_RlmN	Protein synthesis	23S rRNA (adenine(2503)-C(2))-methyltransferase
**TIGR00508**	bioA	Biosynthesis of cofactors, prosthetic groups, and carriers	adenosylmethionine-8-amino-7-oxononanoate transaminase
**TIGR02075**	pyrH_bact	Purines, pyrimidines, nucleosides, and nucleotides	UMP kinase
**TIGR00416**	sms	DNA metabolism	DNA repair protein RadA
**TIGR02386**	rpoC_TIGR	Transcription	DNA-directed RNA polymerase, beta' subunit
**TIGR00651**	pta	Energy metabolism	phosphate acetyltransferase
**TIGR00713**	hemL	Biosynthesis of cofactors, prosthetic groups, and carriers	glutamate-1-semialdehyde-2,1-aminomutase
**TIGR01171**	rplB_bact	Protein synthesis	ribosomal protein uL2
**TIGR00959**	ffh	Protein fate	signal recognition particle protein
**TIGR01455**	glmM	Central intermediary metabolism	phosphoglucosamine mutase
**TIGR01071**	rplO_bact	Protein synthesis	ribosomal protein uL15
**TIGR00952**	S15_bact	Protein synthesis	ribosomal protein uS15
**TIGR01632**	L11_bact	Protein synthesis	ribosomal protein uL11

**TIGR00518**	alaDH	Energy metabolism	alanine dehydrogenase
**TIGR00468**	pheS	Protein synthesis	phenylalanine--tRNA ligase, alpha subunit
**TIGR01736**	FGAM_synth_II	Purines, pyrimidines, nucleosides, and nucleotides	phosphoribosylformylglycinamidine synthase II
**TIGR01737**	FGAM_synth_I	Purines, pyrimidines, nucleosides, and nucleotides	phosphoribosylformylglycinamidine synthase I
**TIGR00420**	trmU	Protein synthesis	tRNA (5-methylaminomethyl-2-thiouridylate)-methyltransferase
**TIGR01066**	rplM_bact	Protein synthesis	ribosomal protein uL13
**TIGR01063**	gyrA	DNA metabolism	DNA gyrase, A subunit
**TIGR02729**	Obg_CgtA	Protein synthesis	Obg family GTPase CgtA
**TIGR00670**	asp_carb_tr	Purines, pyrimidines, nucleosides, and nucleotides	aspartate carbamoyltransferase
**TIGR03263**	guanyl_kin	Purines, pyrimidines, nucleosides, and nucleotides	guanylate kinase
**TIGR03635**	uS17_bact	Protein synthesis	ribosomal protein uS17
**TIGR00184**	purA	Purines, pyrimidines, nucleosides, and nucleotides	adenylosuccinate synthase
**TIGR00343**	TIGR00343	Biosynthesis of cofactors, prosthetic groups, and carriers	pyridoxal 5'-phosphate synthase, synthase subunit Pdx1
