# Supplementary material for: A Human Lung-Associated Streptomyces sp. TR1341 Produces Various Secondary Metabolites Responsible for Virulence, Cytotoxicity and Modulation of Immune Response
Source: Front Microbiol. 2020 Jan 17;10:3028. doi: 10.3389/fmicb.2019.03028 (PMC6978741; doi:10.3389/fmicb.2019.03028)
Supplement: Supplementary file 6 [file Table_4.pdf]

Supplementary Table 4. The results of antibiotic susceptibility of *Streptomyces* sp. TR1341. Means and standard deviations are presented (*n=3*). Results abbreviations – S – sensitive, R - resistant

Antibiotic	Dose	Group	S. sp. TR1341 mean zone (mm)			result
Chloramphenicol	30 µg	amphenicol	20.3	±	2.1	S
Amikacin	30 µg	aminoglycoside	40.7	±	1.5	S
Gentamicin	10 µg	aminoglycoside	38.3	±	1.5	S
Streptomycin	10 µg	aminoglycoside	31.3	±	0.6	S
**Penicillin**	10UI	penicillin	12.7	±	0.6	**R**
Amoxicillin	25 µg	aminopenicillin	21.0	±	1.0	S
**Ampicillin**	10 µg	aminopenicillin	19.0	±	1.0	**R**
Amoxicillin + Clavulanic Acid	20 + 10 µg	aminopenicillin + β-lactamase inhibitor	30.7	±	0.6	S
Cefazolin	30 µg	cephalosporin (1st generation)	21.0	±	0.0	S
**Ceftriaxone**	30 µg	cephalosporin (3rd generation)	17.3	±	1.2	**R**
Vancomycin	30 µg	glycopeptide	25.0	±	0.0	S
**Ciprofloxacin**	5 µg	quinolone (1st generation)	16.3	±	1.5	**R**
**Ofloxacin**	5 µg	quinolone (2nd generation)	14.7	±	1.5	**R**
Erythromycin	15 µg	macrolide (1st generation)	41.3	±	1.2	S
Azithromycin	15 µg	macrolide (2nd generation)	40.3	±	0.6	S
Clarithromycin	15 µg	macrolide (2nd generation)	47.3	±	0.6	S
**Trimethoprim + Sulfamethoxazole**	1,25 µg + 23,75 µg	pyrimidine + sulfonamide	0.0	±	0.0	**R**
**Rifampicin**	5 µg	rifampicin	23.3	±	2.1	**R**
**Tetracycline**	30 µg	tetracycline (1st generation)	15.0	±	0.0	**R**
Doxycycline	30 µg	tetracycline (2nd generation)	21.0	±	1.7	S
Minocycline	30 µg	tetracycline (2nd generation)	24.3	±	0.6	S
